# α-Synuclein-dependent increases in PIP5K1γ drive inositol signaling to promote neurotoxicity

**DOI:** 10.1016/j.celrep.2023.113244

**Published:** 2023-10-14

**Authors:** Jonathan D. Horvath, Maria Casas, Candice Kutchukian, Sara Creus Sánchez, Melissa R. Pergande, Stephanie M. Cologna, Sergi Simó, Rose E. Dixon, Eamonn J. Dickson

**Affiliations:** 1 Department of Physiology and Membrane Biology, University of California, Davis, Davis, CA 95616, USA; 2 Department of Chemistry, University of Illinois, Chicago, IL, USA; 3 Department of Cell Biology and Human Anatomy, University of California, Davis, Davis, CA 95616, USA; 4 Lead contact

## Abstract

Anomalous aggregation of α-synuclein (α-Syn) is a pathological hallmark of many degenerative synucleinopathies including Lewy body dementia (LBD) and Parkinson’s disease (PD). Despite its strong link to disease, the precise molecular mechanisms that link α-Syn aggregation to neurodegeneration have yet to be elucidated. Here, we find that elevated α-Syn leads to an increase in the plasma membrane (PM) phosphoinositide PI(4,5)P_2_, which precipitates α-Syn aggregation and drives toxic increases in mitochondrial Ca^2+^ and reactive oxygen species leading to neuronal death. Upstream of this toxic signaling pathway is PIP5K1γ, whose abundance and localization is enhanced at the PM by α-Syn-dependent increases in ARF6. Selective inhibition of PIP5K1γ or knockout of ARF6 in neurons rescues α-Syn aggregation and cellular phenotypes of toxicity. Collectively, our data suggest that modulation of phosphoinositide metabolism may be a therapeutic target to slow neurodegeneration for PD and other related neurodegenerative disorders.

## In brief

α-Synuclein (α-Syn) aggregation is a key feature of neurodegenerative disorders like Lewy body dementia and Parkinson’s disease. Horvath et al. demonstrate that α-Syn fibrils or disease mutations increase the membrane phosphoinositide PI(4,5)P_2_, which influences α-Syn aggregation and leads to increases in mitochondrial Ca^2+^ and reactive oxygen species, causing neuronal death.

## Graphical Abstract

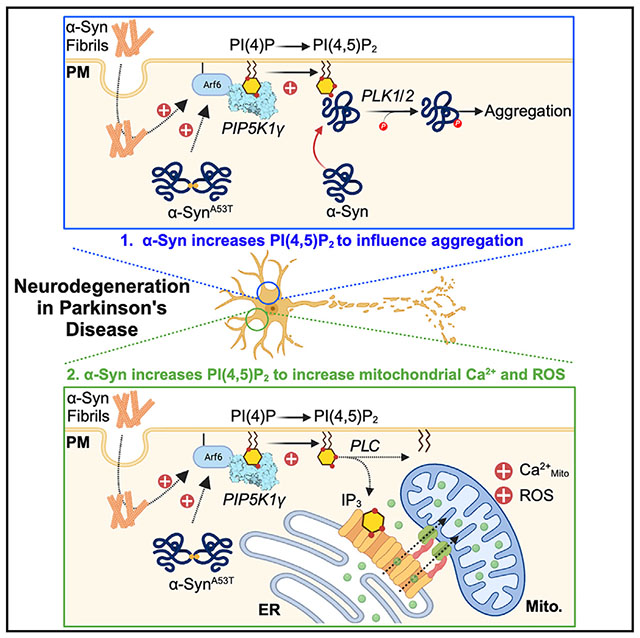

## INTRODUCTION

α-Synuclein (α-Syn) is one of three members of the Syn family (α-, β-, and γ-) that is encoded by the *SNCA* gene and is abundantly expressed in neuronal tissues. A small (140-residue), natively unfolded protein, α-Syn was initially observed to localize in the nucleus and presynaptic terminals of neurons.^1^ Based on its presynaptic localization, most of α-Syn’s physiological roles have centered around its ability to influence neurotransmission and synaptic vesicle dynamics^2–6^; however, it is clear that α-Syn can also affect signaling reactions and membrane function of many organelles including the endoplasmic reticulum (ER),^7–9^ Golgi,^7,10^ mitochondria,^11,12^ and endo-/lysosomes.^13–15^ Pathologically, missense mutations in *SNCA* lead to enhanced α-Syn aggregation and cause inherited forms of Parkinson’s disease (PD).^16–20^ Idiopathically, α-Syn aggregation leads to the formation of toxic α-Syn fibrils that constitute the building blocks of Lewy bodies, the deviant protein deposits that accumulate and propagate between neurons, leading to Lewy body dementia and PD.^21–24^ Thus, from a genetic and idiopathic perspective, increased aggregation of α-Syn is a key pathological hallmark. Despite clear neuropathological consequences for α-Syn aggregation, there is a lack of mechanistic information regarding the intracellular pathways that influence α-Syn aggregation to promote neuronal demise.^25^

The local membrane lipid environment has been suggested to play an important role in the aggregation of α-Syn,^26–29^ with membrane bound α-Syn having a higher propensity to seed aggregation of the more abundant, cytosolic form of α-Syn.^30^ α-Syn localizes to membrane-cytosol interfaces through interactions between its amphipathic N-terminal domain^31^ and acidic phospholipids.^32^ Among lipids reported to interact with α-Syn are phosphoinositides,^27,33–35^ a family of low-abundance, negatively charged phospholipids whose inositol ring can be phosphorylated or dephosphorylated at hydroxyl positions 3, 4, or 5 to generate seven polyphosphoinositide (PPI) species from the parent PI molecule. The signature PPI species of the plasma membrane (PM) is PI(4,5)P_2_; here, it is crucial for the control of a wide range of cellular processes, including endo- and exocytosis, which have been linked to the pathogenic spread of α-Syn.^22,34,36,37^ The majority of PI(4,5)P_2_ is produced through the enzymatic actions of type I PIP kinases (PIPK1α, -β, and -γ) on precursor PI(4)P pools. PIP5K1γ is the major isoform expressed in neuronal tissue and highly concentrated at synapses.^38^ Linking PM PI(4,5)P_2_ to PD are mutations in the PI(4,5)P_2_-metabolizing enzyme synaptojanin1 (SYNJ1) that cause inherited forms of PD (PARK20)^39^ and SYJN1 haploinsufficiency, which drives dopaminergic neuron vulnerability.^40^ Despite these correlative associations, there remains a paucity of information on the molecular underpinnings that link α-Syn aggregation, PI(4,5)P_2_ dysregulation, and neuronal cell death.

In this present study, we determine that treatment of neurons with preformed human α-Syn fibrils or expression of disease mutations in α-Syn increase PM PI(4,5)P_2_ through enhanced ARF6-dependent recruitment of PIP5K1γ. Elevations in PM PI(4,5)P_2_ (1) seeds aggregation of cytosolic α-Syn and (2) potentiates ER-mediated IP_3_ Ca^2+^ release to drive neurotoxic elevations in mitochondrial Ca^2+^ levels. Inhibition or knockdown of PIP5K1γ, or other key regulators of this signaling pathway, normalizes PM PI(4,5)P_2_, decreases α-Syn aggregates and rescues neuron viability. These data suggest that PM PI(4,5)P_2_ plays a crucial role in the pathological progression of PD and that modulation of enzymes that regulate PI(4,5)P_2_ metabolism may provide a therapeutic target to treat and ameliorate the devastating consequences of PD or other synucleinopathies.

## RESULTS

### α-Syn disease mutations or human α-Syn fibrils increase PM PI(4,5)P_2_ across different brain regions

Increased α-Syn aggregation and fibrillar formation represent key pathological hallmarks of PD and Lewy body dementias.^41–44^ It is for this reason that throughout the present study, we use (1) α-Syn^A53T^, a familial PD mutation that exhibits an increased propensity to aggregate^24,45^ (Figure S1A), and (2) human preformed α-Syn fibrils (referred to hereafter as α-Syn fibrils), a model of synucleinopathy that seeds recruitment and aggregation of endogenous α-Syn, leading to neuronal dysfunction and degeneration^24,42,46^ (Figure S1B). Collectively, these pathological cell models allow us to investigate if common molecular mechanisms precipitate neurodegeneration across two distinct models of PD and related dementias.

**Figure 1.**
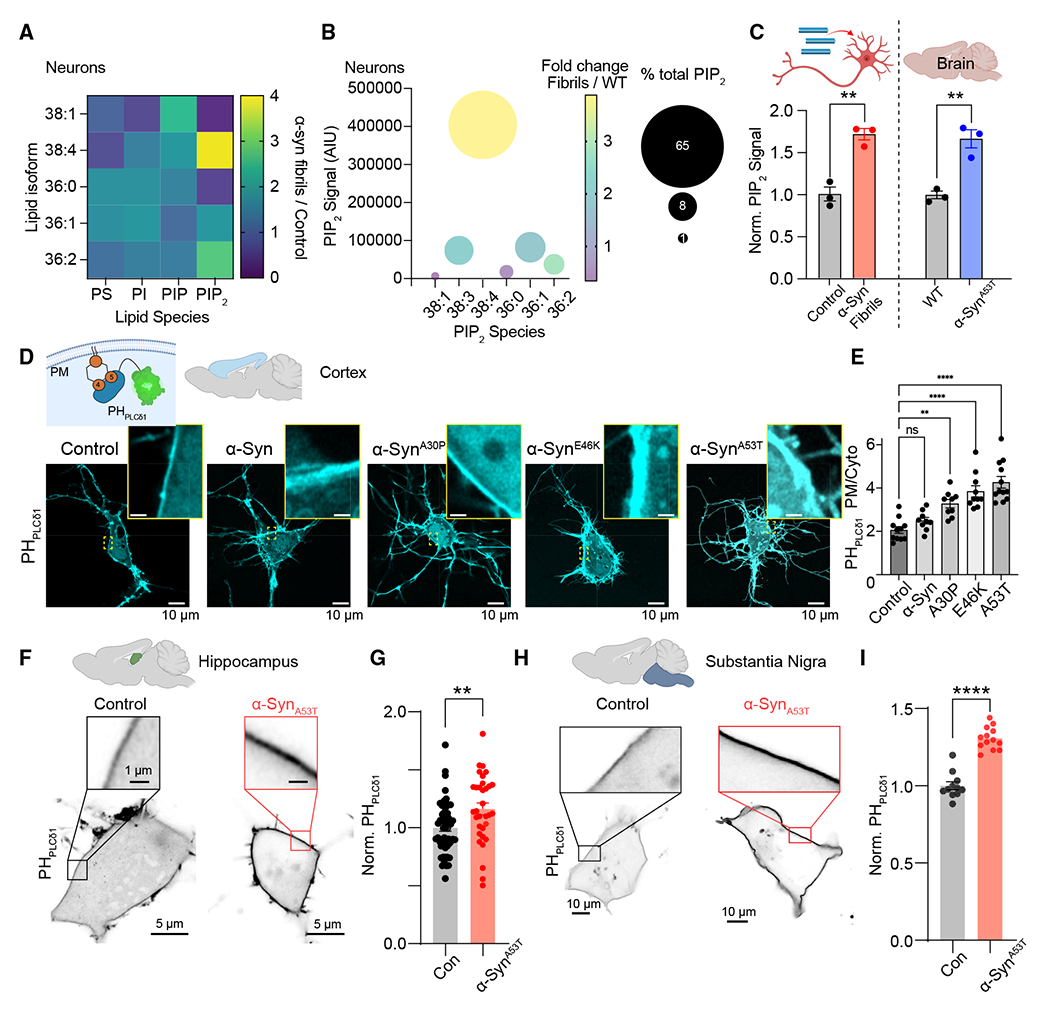
Treatment with α-Syn fibrils or α-Syn Parkinson’s disease mutations increase plasma membrane PI(4,5)P_2_ across different brain regions (A) Heatmap presenting fold change of different phospholipid species (and their isoforms) from isolated neurons treated for 14 days with α-Syn fibrils. (B) Bubble chart presenting the absolute and fold change in each PIP_2_ species from isolated neuron cultures treated with PBS control or α-Syn fibrils. (C) Lipid mass spectrometry (UHPLC-MS/MS) analysis detailing changes in total PIP_2_ levels measured from isolated neurons treated with α-Syn fibrils or brains from α-Syn^A53T^ mice relative to controls. Statistical analysis was a Student’s t test. (D) Inset: diagram detailing the binding of PH_PLCδ1_ to PI(4,5)P_2_ at the plasma membrane (PM). Representative confocal micrographs of cortical neurons expressing PH_PLCδ1_ and either monomeric α-Syn or α-Syn Parkinson’s disease mutations (α-Syn^A30P^, α-Syn^E46K^, α-Syn^A53T^). (E) One-way ANOVA analysis of PH_PLCδ1_ distribution between the PM and cytoplasm under conditions of altered α-Syn expression. (F) Representative confocal images showing PH_PLCδ1_ localization in control and α-Syn^A53T^-transfected hippocampal neurons. (G) Quantification of normalized PM/cytoplasm intensity of PH_PLCδ1_ in mouse hippocampal neurons. Statistical analysis was a Student’s t test. (H and I) Same as (F) and (G) only for substantia nigra neurons. Error bars represent the standard error of the mean. NS, not significant; **p < 0.01; ****p < 0.0001.

To begin, we performed ultra-high-performance liquid chromatography coupled with tandem mass spectrometry (UHPLC-MS/MS) to quantify absolute changes in several phospholipid species from two models of PD. First, by treating isolated neurons with human preformed α-Syn fibrils, we determined that although there were differential abundances of several phosphatidylserine (PS) species (Figure 1A), total brain PS was unaltered (Figure S1C). Similarly, levels of phosphatidylinositol (PI) and phosphatidylinositol monophosphate (PIP) showed heterogeneity in abundance between species (Figure 1A) but no change in total levels (Figure S1C). In comparison with PS, PI, and PIP, investigation of individual phosphatidylinositol bisphosphate (PIP_2_) species revealed that in addition to several species being significantly elevated (Figures 1A and 1B), including PIP_2_ 38:4, which represents the most abundant species in primary cells,^47^ total PI(4,5)P_2_ exhibited a >50% increase in levels relative to control (Figure 1C). Like treatment of isolated neurons with α-Syn fibrils, total brain PIP_2_ levels from a mouse model expressing mutant human α-Syn^A53T 24^ were also significantly elevated relative to age- and sex-matched wild-type animals (Figure 1C). Thus, α-Syn fibrils and α-Syn^A53T^ PD mutation increase cellular PI(4,5)P_2_.

MS analysis of PIP_2_ levels is extremely quantitative but does not provide (1) spatial information regarding intracellular pools of PIP_2_ or (2) which PIP_2_ species (PI(4,5)P_2_, PI(3,4)P_2_, or PI(3,5)P_2_) is increased following increases in α-Syn expression. Given the strong link between altered PI(4,5)P_2_ and PD^39,40^ and that PI(4,5)P_2_ represents >80% of the total cellular PIP_2_ pool,^48^ we quantified the intracellular distribution of PI(4,5)P_2_ using the genetically encoded PI(4,5)P_2_ biosensor PH_PLCδ1_.^49^ To begin, we expressed PH_PLCδ1_ in isolated cortical neurons and quantified its distribution under control conditions, conditions of overexpressed monomeric α-Syn, or conditions of overexpressed α-Syn PD mutations (Figure 1D). We choose different PD mutations to determine if increases in PI(4,5)P_2_ were selective for a single α-Syn mutation or were a common observation across multiple familial PD mutations. Analysis revealed that overexpression of monomeric α-Syn did not significantly alter PH_PLCδ1_ distribution, whereas α-Syn^A30P^, α-Syn^E46K^, and α-Syn^A53T^ all significantly increased PH_pLCδ1_ at the PM (Figures 1D and 1E). To confirm that α-Syn can increase PI(4,5) P_2_ across multiple brain regions, we expressed PH_PLCδ1_ in isolated neurons from two different brain regions and found that PH_PLCδ1_ increased in the PM of hippocampal (Figures 1F and 1G) and substantia nigra (Figures 1H and 1I) neurons following expression of α-Syn^A53T^. Similar increases in steady-state PM PH_PLCδ1_ were observed in CHO cells and fibroblasts from patients with PD (Figures S1E and S1F). Unlike PH_PLCδ1_, α-Syn^A53T^ did not alter PI(3)P or PI(3,4,5)P_3_ biosensor distributions (Figures S1F and S1G). Finally, to confirm that increases in PI(4,5)P_2_ were selective for α-Syn aggregation and not a general phenomenon of pathological protein aggregates, we quantified PH_PLCδ1_ distribution with or without treatment with tau or amyloid-β (Aβ) fibrils. Consistent with published literature, neither tau nor Aβ fibrils increased PM PI(4,5)P_2_ (Figure S1H).^50,51^ Taken together, these data determine that PM PI(4,5)P_2_ levels are increased in brain tissue and isolated neuronal and non-neuronal cells following overexpression of α-Syn disease mutations or treatment with α-Syn fibrils.

### PIP5K1γ drives α-Syn-dependent increases in PI(4,5)P_2_

**Figure 2.**
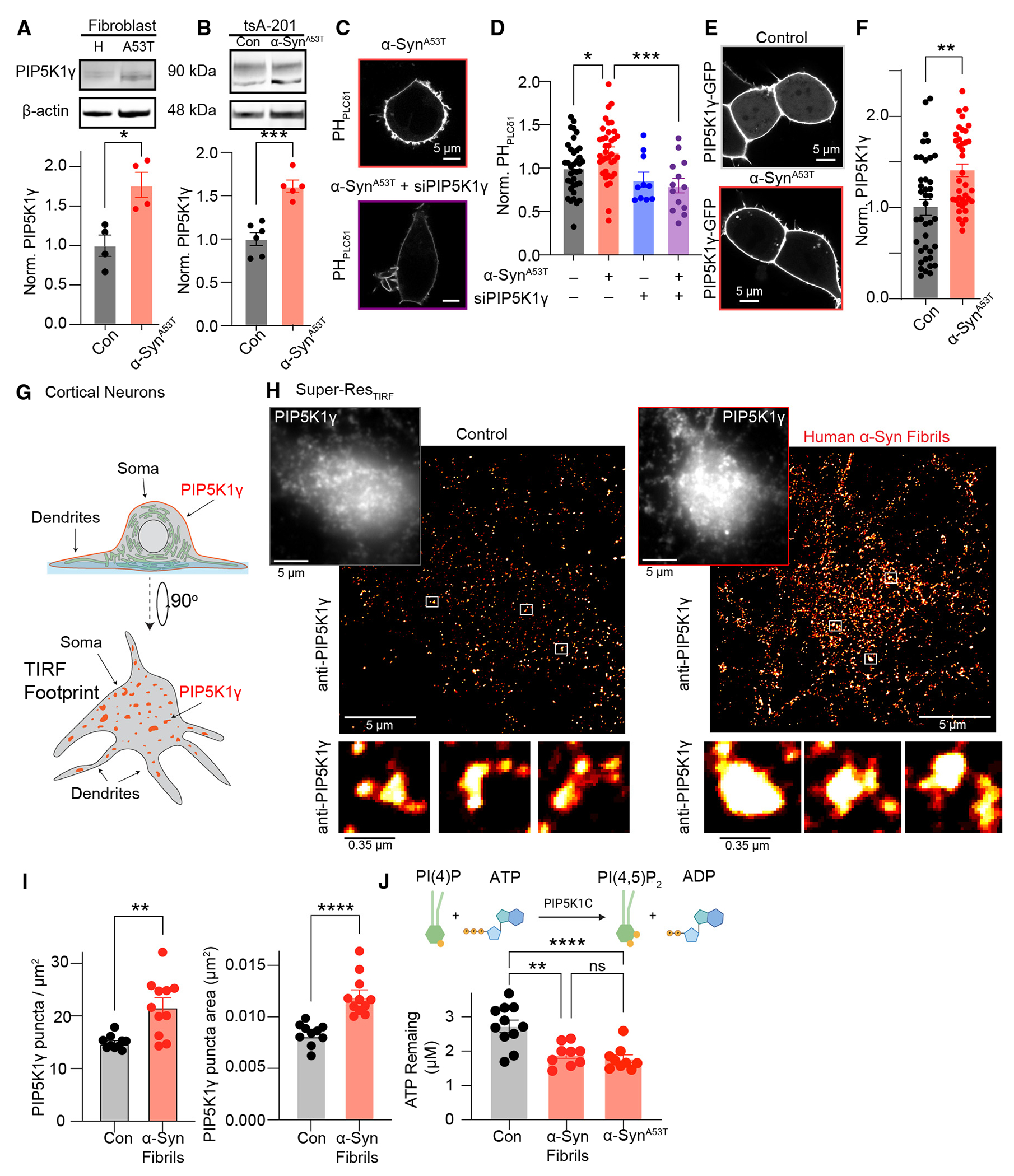
Increased expression, distribution, and activity of PIP5K1γ drives α-Syn-dependent increases in PI(4,5)P_2_ (A) Top: representative western blot of PIP5K1γ and β-actin from control and α-Syn^A53T^ patient fibroblasts. Bottom: quantification of PIP5K1γ normalized to β-actin. Statistical analysis was a Student’s t test. (B) Same as (A) but only HEK293T cells expressing α-Syn^A53T^. Statistical analysis was a Student’s t test. (C) Representative images of α-Syn^A53T^ and PH_PLCδ1_-CFP-transfected HEK293T cells transfected with or without siRNA targeting PIP5K1γ. (D) Quantification of normalized PM/cytoplasm intensity of PIP5K1γ. Statistical analysis was a two-way ANOVA. (E) Representative confocal images of GFP-PIP5K1γ from control and α-Syn^A53T^-transfected HEK293T cells. (F) Quantification of GFP-PIP5K1γ distribution at the PM relative to cytoplasm. Statistical analysis was a Student’s t test. (G) Schematic of TIRF imaging for PIP5K1γ in mouse neurons. (H) Representative TIRF images (diffraction limited and super-resolution) of control and α-Syn fibril-treated mouse cortical neurons stained for PIP5K1γ. (I) Quantification of super-resolution TIRF images. Statistical analyses were Student’s t tests. (J) Top: diagram of assay. Bottom: histogram quantifying the concentration of ATP remaining in neuron samples from control, treated with α-Syn fibrils, or expressing α-Syn^A53T^. Error bars represent the standard error of the mean. ns, not significant; *p < 0.05; **p < 0.01; ***p < 0.001; ****p < 0.0001.

Elevations in PI(4,5)P_2_ observed following α-Syn fibril treatment or overexpression of α-Syn^A53T^ prompted us to ask what molecular mechanisms were responsible for these changes. PM PI(4,5) P_2_ levels are controlled by the actions of multiple lipid kinases and phosphatases. In neurons, PIP5K1γ enzymatic activity on precursor PI(4)P pools represents the major route of PI(4,5)P_2_ production.^52^ With this in mind, we wanted to understand if changes in α-Syn^A53T^ expression or treatment with α-Syn fibrils alter the distribution, abundance, and/or activity of PIP5K1γ. To begin, we performed western blot experiments and determined that fibroblasts harboring the α-Syn^A53T^ mutation and HEK293T cells overexpressing α-Syn^A53T^ exhibited small, but statistically significant, elevations in PIP5K1γ protein levels compared with controls (Figures 2A and 2B). The expression of two other important PI(4,5)P_2_-metabolizing enzymes, PIP5K1α or SYNJ1, were unchanged or increased, respectively, following α-Syn fibril treatment or α-Syn^A53T^ overexpression (Figures S2A and S2B), suggesting that increases in PI(4,5)P_2_ likely occur through PIP5K1γ. Supporting this hypothesis, inhibition of PIP5K1α with ISA-2011B did not reduce α-Syn^A53T^ increases in PI(4,5)P_2_ (Figure S2C), whereas small interfering RNA (siRNA)-mediated knockdown of PIP5K1γ reduced stead-state PI(4,5) P_2_ levels and resulted in cells being refractory to α-Syn-dependent increases in PI(4,5)P_2_ (Figures 2C and 2D).

PIP5K1 γ is a cytosolic protein that is recruited to cellular membranes to catalyze the production of PI(4,5)P_2_; therefore, we also wanted to test if its localization was altered under different α-Syn conditions. Using AiryScan super-resolution confocal microscopy, we expressed GFP-PIP5K1γ with or without α-Syn^A53T^ in HEK293T cells and discovered that, in control cells, GFP-PIP5K1γ is localized to both the PM and cytoplasm, with cotransfection of α-Syn^A53T^ shifting its distribution toward the PM (Figures 2E and 2F). To test if all PI(4,5)P_2_-influencing proteins increase at the PM, we expressed the PI transfer protein Nir2 and its binding partner VAPA and found that it was insensitive to changes in α-Syn (Figure S2D), as was SYNJ1 (Figure S2E), suggesting that α-Syn selectively increases PM PI(4,5)P_2_ through PIPK1γ. To test if α-Syn alters endogenous PIP5K1γ distribution at the PM, we used a validated PIP5K1γ-antibody^38^ and conducted super-resolution total internal reflection fluorescence (TIRF) imaging experiments in neurons to visualize the enzymes’ distribution close to the PM. Quantitative analysis of singlemolecule localization microscopy maps (resolution ~20–30 nm^53–57^) determined that both the number and the area of PIP5K1γ puncta were significantly increased when neurons were cultured with α-Syn fibrils relative to PBS control (Figures 2G–2I). Finally, to determine if enhanced PM recruitment of PIP5K1γ altered its catalytic activity, we performed PIP5K1 ATP depletion assays and found that lysates from neurons treated with α-Syn fibrils or expressing α-Syn^A53T^ had less ATP remaining relative to control samples, suggesting enhanced enzymatic activity of PIP5K1γ (Figure 2J). Collectively, these data provide evidence that the PM localization and the activity of PIP5K1γ are increased under conditions of α-Syn fibril treatment or overexpression of α-Syn^A53T^ PD mutations.

### ARF6-dependent recruitment of PIP5K1γ underlies α-Syn increases in PI(4,5)P_2_

**Figure 3.**
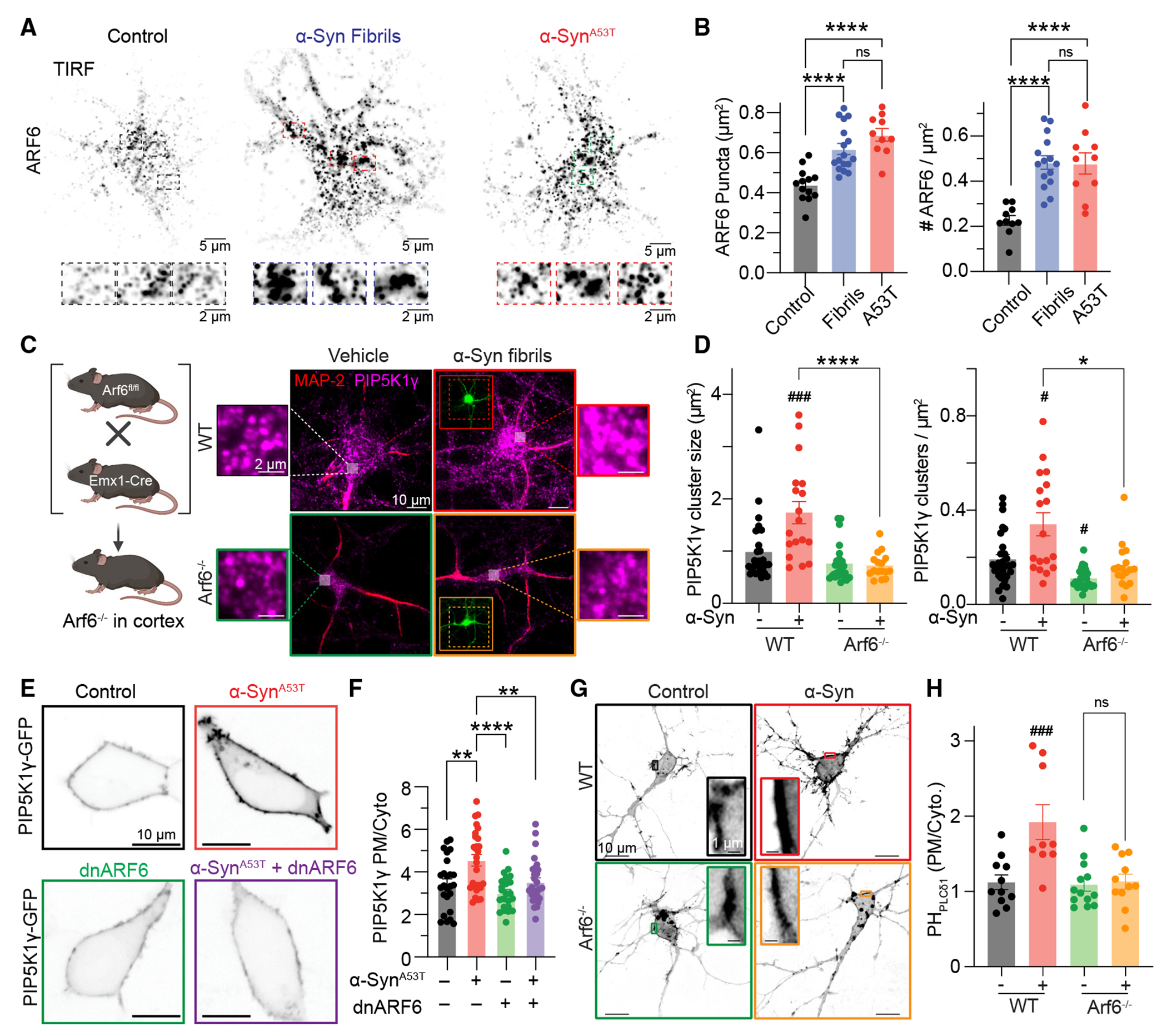
ARF6-dependent recruitment of PIP5K1γ underlies α-Syn increases in PI(4,5)P_2_ (A) Top: representative TIRF images from neurons fixed and stained for ARF6 under control (left), α-Syn fibril treatment (middle), or α-Syn^A53T^ expression. Bottom: enlarged regions of insert from black dashed rectangles. (B) Quantification of ARF6 puncta size (left) and density (right). Statistical analysis was one-way ANOVA. (C) Left: strategy for knocking out ARF6 (*ARF6*^−/−^) in the telencephalon. Right: representative TIRF images from WT and *ARF6*^−/−^ neurons treated with or without α-Syn fibrils fixed and stained for ARF6. (D) Quantification of PIP5K1γ size (left) and density (right). Statistical analysis was two-way ANOVA. (E) Representative confocal micrographs for HEK293T cells expressing GFP-PIP5K1γ with either α-Syn^A53T^, DNARF6, or α-Syn^A53T^ and DNARF6. (F) Quantification of GFP-PIP5K1γ distribution. Statistical analysis was two-way ANOVA. (G) Representative confocal images of WT and *ARF6*^−/−^ neurons expressing PH_PLCδ1_ with or without α-Syn fibril treatment. (H) Quantification of PH_PLCδ1_ distribution. Statistical analysis was one-way ANOVA. Error bars represent the standard error of the mean. #, significantly different from WT (p < 0.05); ###, significantly different from WT (p < 0.001); ns, not significant; *p < 0.05; **p < 0.01; ***p < 0.001; ****p < 0.0001.

PIP5K1γ is recruited to the PM through interactions with ADP-ribosylation factor and small GTPase ARF6.^58^ A recent MS proteomics investigation to determine proteins differentially regulated by α-Syn fibrils identified ARF6 as being significantly elevated 14 days post-treatment.^59^ To investigate an upstream role for ARF6 in facilitating the recruitment of PIP5K1γ under conditions of α-Syn fibrils or α-Syn^A53T^ mutation, we fixed and stained isolated neurons for ARF6 and characterized its localization close to the PM using TIRF microscopy. In control conditions, TIRF imaging revealed a heterogeneous punctate distribution of ARF6 within the TIRF footprint aligned with its role in recruiting PIP5K1γ to make PI(4,5)P_2_ (Figure 3A). Treatment of neurons with α-Syn fibrils or expression of α-Syn^A53T^ increased the size and density of ARF6 within the TIRF footprint (Figures 3A and 3B). To directly test for a role of ARF6 influencing recruitment of PIP5K1γ to the PM, we took a genetic approach to knock out ARF6 in the cortex by crossing ARF6^fl/fl^ and Emx1-Cre mice.^60^ TIRF imaging of isolated neurons revealed that while treatment of wild-type (WT) neurons with α-Syn fibrils enhanced recruitment of PIP5K1γ to the PM, *ARF6*^−/−^ neurons were completely refractory (Figures 3C and 3D). To test if α-Syn^A53T^ also recruits PIP5K1γ through an ARF6-dependent mechanism, we expressed α-Syn^A53T^ with or without a dominant-negative ARF6 (dnARF6) and monitored GFP-PIP5K1γ distribution. We found that treatment with α-Syn^A53T^ enhanced GFP-PIP5K1γ at the PM, whereas expression of α-Syn^A53T^ and dnARF6 abrogated recruitment (Figure 3E). Finally, to confirm that increases in PI(4,5)P_2_ observed following treatment with α-Syn fibrils (Figure 1A) occur through ARF6-mediated recruitment of PIP5K1γ, we expressed the PI(4,5)P_2_ biosensor (PH_PLCδ1_) in WT and *ARF6*^−/−^ neurons and quantified its distribution. Analysis of confocal micrographs determined that increases in PM PH_PLCδ1_ observed following α-Syn fibril treatment were completely absent in *ARF6*^−/−^ neurons (Figures 3G and 3H). These data support the hypothesis that α-Syn fibrils and disease mutations in α-Syn increase PI(4,5)P_2_ through ARF6-dependent recruitment of PIP5K1γ.

### ARF6-PIP5K1γ-dependent increases in PI(4,5)P_2_ influence α-Syn aggregation

**Figure 4.**
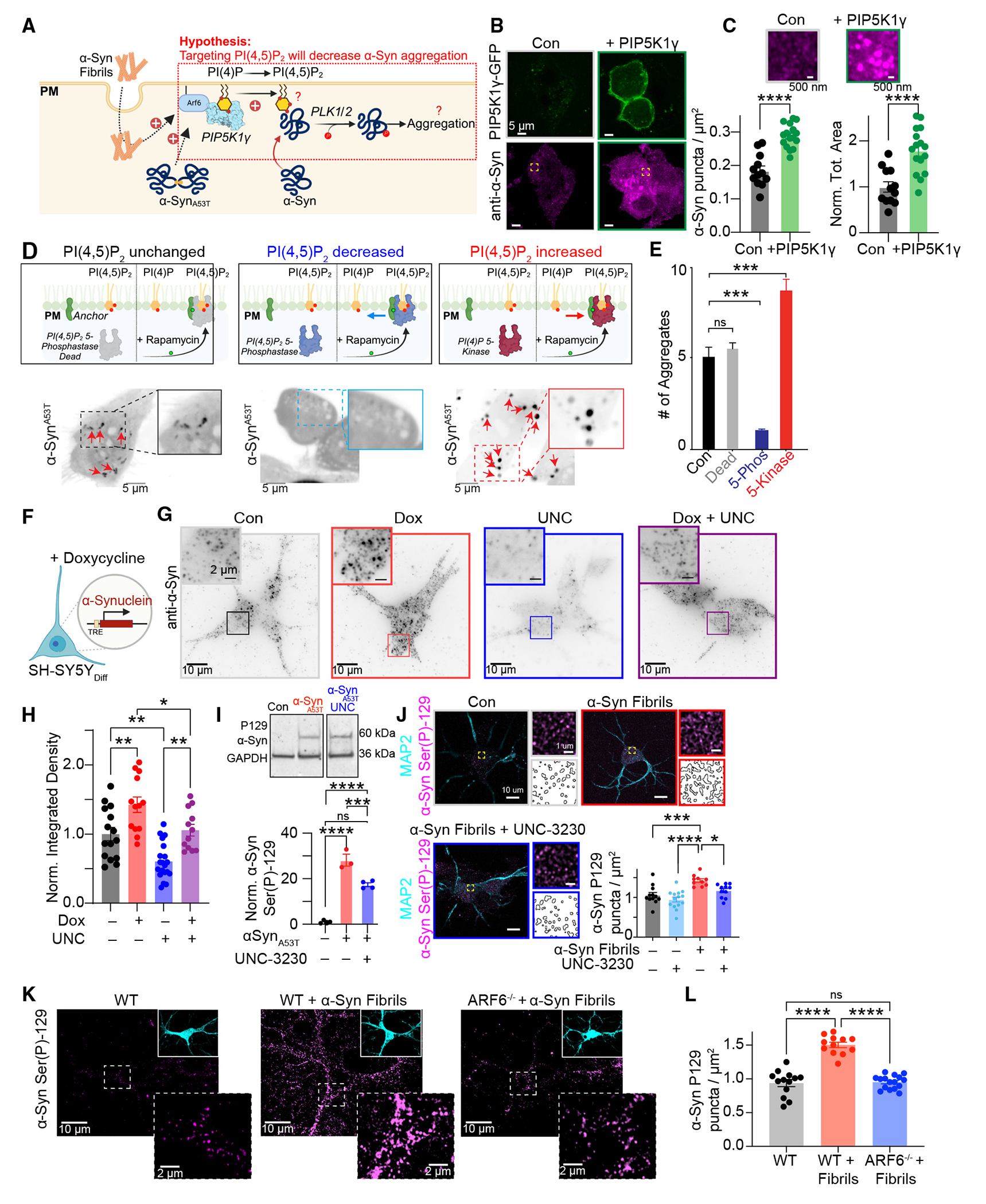
ARF6-PIP5K1γ signaling axis influences α-Syn aggregation (A) Schematic of hypothesis: targeting PI(4,5)P_2_ can decrease α-Syn aggregation. (B) HEK293T cells fixed and stained for α-Syn with or without GFP-PIP5K1γ expression. (C) Quantification of α-Syn density (left) and total area (right) under control or PIP5K1γ-expressing conditions. Statistical analysis was students t-test. (D) Top: schematic of rapamycin-induced dimerization for each condition. Bottom: representative TIRF images of α-Syn^A53T^-GFP from HEK293T cells following recruitment of a phosphatase dead (left), PI(4,5)P_2_ 5-phosphatase (middle), and PI(4)P 5-kinase (right). (E) Quantification of number of α-Syn^A53T^-GFP within the TIRF footprint. (F) Left: diagram illustrating doxycycline-dependent increases in α-Syn. (G) Representative TIRF images of α-Syn^Dox^ cells fixed and stained for α-Syn under conditions of DMSO control, doxycycline, UNC3230, or doxycycline and UNC3230. (H) Quantification of α-Syn integrated density within the TIRF footprint intensity. Statistical analysis was two-way ANOVA. (I) Top: representative western blot of P129-α-Syn. Bottom: quantification of P129-α-Syn from each condition normalized to GAPDH. (J) Representative TIRF images from neurons fixed and stained of MAP2 and P129-α-Syn under different conditions. Statistical analysis was two-way ANOVA. (K) Representative TIRF images of WT and *ARF6*^−/−^ neurons treated with monomeric α-Syn or α-Syn fibrils fixed and stained for α-Syn. Inserts show expression of monomeric α-Syn and α-Syn fibrils. (L) Analysis of α-Syn density. Statistical analysis was one-way ANOVA. Error bars represent the standard error of the mean. ns, not significant; *p < 0.05; **p < 0.01; ***p < 0.001; ****p < 0.0001.

Pathologically, the binding of α-Syn to negatively charged phosphoinositide lipid membranes has been proposed as a key proximal step in the toxic cascade of α-Syn aggregation.^61,62^ Furthermore, α-Syn has been shown to positively correlate with PI(4,5)P_2_ levels at cellular membranes.^35^ Since α-Syn fibril treatment or α-Syn^A53T^ favor ARF6-dependent recruitment of PIP5K1γ to increase PI(4,5)P_2_, we wanted to determine if this signaling pathway influences α-Syn aggregation at the PM (Figure 3A). To begin, we overexpressed PIP5K1γ and fixed and stained for endogenous α-Syn in HEK293T cells. Quantitative analysis from the resulting super-resolution images revealed that overexpression of GFP-PIP5K1γ increased both the density and the area of α-Syn puncta (Figures 4B and 4C). Next, to see if PI(4,5)P_2_ is required for α-Syn aggregation at the PM, we utilized a rapamycin-induced dimerization system to increase or decrease the levels of PI(4,5)P_2_ under conditions of α-Syn^A53T^ expression^63^ (Figure 4D). In control experiments, recruitment of a kinase-dead mutant, which lacks the ability to dephosphorylate PI(4,5)P_2_, resulted in no significant changes to α-Syn^A53T^ aggregation near the PM (Figures 4D and 4E). In comparison, recruitment of a PI(4,5)P_2_ 5-phosphatase to decrease PM PI(4,5)P_2_^55,64,65^ reduced α-Syn^A53T^ aggregates, whereas recruitment of a PI(4)P 5-kinase to increase PM PI(4,5)P_2_ increased α-Syn^A53T^ aggregates (Figures 4D and 4E). These experiments suggest that PM PI(4,5)P_2_, but not its precursor PI(4)P, is a key factor that influences the ability of α-Syn to form aggregates at the PM. To further test this model, we used a doxycycline-inducible SH-SY5Y cell line that allows for increased expression of α-Syn with addition of doxycycline^66^ (hereafter called α-Syn^Dox^ cells; Figure 4F). α-Syn^Dox^ cells were cultured with vehicle control, doxycycline (to increase α-Syn expression and aggregate formation near the PM; Figures 4G, S3A, and S3B), the selective PIP5K1γ inhibitor UNC3230,^67^ or doxycycline in the presence of UNC3230 before being fixed and stained for α-Syn. Analysis of the resulting TIRF footprints revealed that increased expression of cellular α-Syn increased its aggregation at the PM (Figures 4G and 4H, red), with UNC3230 cotreatment, to decrease PM PI(4,5)P_2_, normalizing α-Syn levels back into a control range (Figures 4G and 4H, purple). To more directly test if PM PI(4,5)P_2_ levels act as an upstream rheostat for α-Syn aggregation at membranes, we blotted for phoso-S129 α-Syn, a phosphorylation site that enhances membrane binding and α-Syn toxicity.^68^ Analyses revealed that overexpression of α-Syn^A53T^ increased S129 α-Syn, while inhibition of PIP5K1γ significantly decreased its total cellular abundance (Figure 4I). Similar results were observed using multiplexed imaging approaches (Figure 4J). These data suggest that PM PI(4,5)P_2_ acts a molecular scaffold to recruit α-Syn to the PM to control its phosphorylation and aggregation status. Finally, to test if ARF6 is upstream of PIP5K1γ and part of the signaling pathways that lead to increased α-Syn aggregation, we performed experiments using *ARF6*^−/−^ neurons and found that loss of ARF6 function abrogated S129 α-Syn aggregation (Figures 4K and 4L). We propose that α-Syn fibrils facilitate ARF6-dependent recruitment of PIP5K1γ to catalyze the production of PI(4,5)P_2_ and the seed aggregation of monomeric α-Syn at the PM in models of PD.

### α-Syn-dependent increases in PI(4,5)P_2_ enhance IP_3_R1 clustering to augment G_q_-mediated Ca^2+^ release

**Figure 5.**
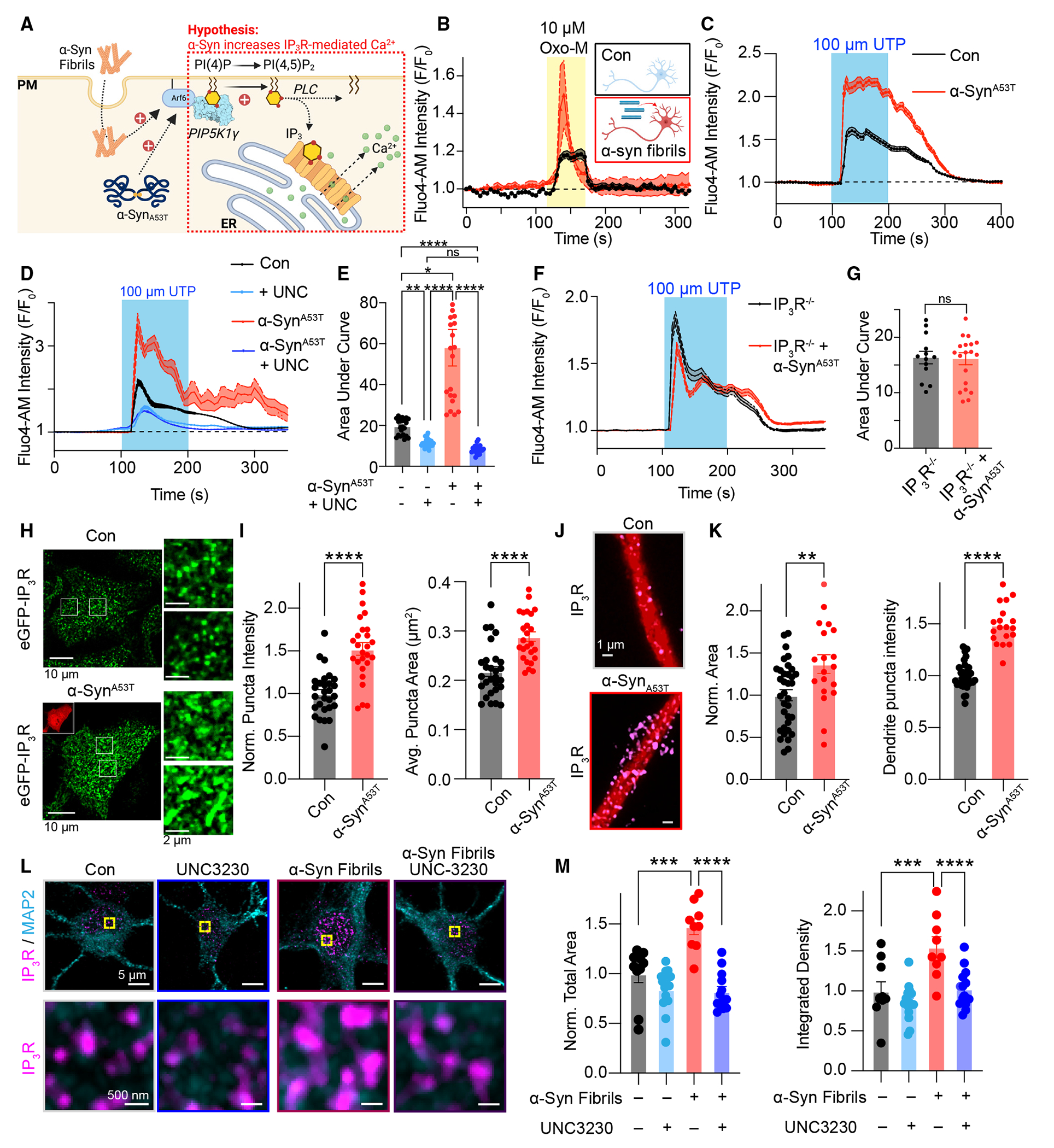
α-Syn augments IP_3_-mediated Ca^2+^ release (A) Schematic of hypothesis to be tested: α-Syn-dependent increases in PI(4,5)P_2_ influence IP_3_-mediated ER Ca^2+^ release. (B) Representative time series of Fluo-4 AM responses from neurons treated with PBS control or α-Syn fibrils and treated with Oxo-M (10 μM). (C) Quantification of normalized Fluo-4 AM intensity in control and α-Syn^A53T^-transfected HEK293T cells during UTP application. (D and E) Quantification of normalized Fluo-4 AM intensity in control and α-Syn^A53T^-transfected HEK293T cells with or without treatment with 100 nM UNC-3230 during UTP perfusion. Statistical analysis was a two-way ANOVA. (F and G) Quantification of normalized Fluo-4 AM intensity in control and α-Syn^A53T^ -transfected IP_3_R1 knockout HEK293 cells during UTP application. Statistical analysis was a Student’s t test. (H) Representative confocal images of control and α-Syn^A53T^-transfected eGFP-IP_3_R1 cells. (I) Quantification of size and intensity of IP_3_R1 puncta. Statistical analysis was a Student’s t test. (J) Representative confocal images of control and α-Syn^A53T^-transfected neurons with MAP2-labeled dendrites (red) stained for IP_3_R1 (magenta). (K) Quantification of size (left) and intensity (right) of IP_3_R puncta in hippocampal dendrites. Statistical analysis was a Student’s t test. (L) Representative confocal images of control and α-Syn fibril-treated mouse neurons stained with IP_3_R1 (magenta) and MAP2 (cyan) with or without treatment of 100 nM UNC-3230. Zoomed-in images show only IP_3_R1. (M) Quantification of area (left) and integrated density (right) of IP_3_R1 in neurons with or without α-Syn or UNC3230 treatment. Statistical analyses were two-way ANOVAs. Error bars represent the standard error of the mean. ns, not significant; **p < 0.01; ***p < 0.001; ****p < 0.0001.

Having established that α-Syn fibrils, a key component of Lewy bodies, increase PM PI(4,5)P_2_ to seed α-Syn phosphorylation and aggregation, we next sought to determine if α-Syn-dependent elevations in PI(4,5)P_2_ also influenced another PM PI(4,5) P_2_-dependent event. We focused on IP_3_-mediated Ca^2+^ signaling for the following reasons: (1) PI(4,5)P_2_ is the precursor for IP_3_ generation, (2) cytoplasmic Ca^2+^ concentrations are thought to positively influence α-Syn aggregation,^69,70^ and (3) IP_3_-mediated Ca^2+^ signaling is a driver of cell death in other neurodegenerative disorders.^71–74^ Briefly, binding of neurotransmitters or hormones to G_q_PCRs leads to activation of phospholipase C (PLC), hydrolysis of PI(4,5)P_2_, and subsequent production of cytosolic IP_3_ and membrane-bound DAG. Diffusible IP_3_ is then free to bind IP_3_ receptors (IP_3_Rs) in ER membranes to initiate release of Ca^2+^ into the cytoplasm, which serves as an instructional signal to alter the activity of Ca^2+^-sensitive proteins (Figure 5A). Based on the finding that α-Syn increases cellular PI(4,5)P_2_ levels (Figure 1) and the critical role of PIP5K1γ in driving IP_3_-mediated Ca^2+^ release,^75^ we wanted to test if G_q_PCR signaling is altered under conditions of enhanced pathological α-Syn.

To ascertain the potential impact of α-Syn fibrils on G_q_-mediated Ca^2+^ release dynamics, neurons were subjected to either a control medium (PBS) or α-Syn fibrils for a duration of 14 days. Following incubation, neurons were loaded with the Ca^2+^-sensitive indicator Fluo-4 AM, and changes in cytosolic Ca^2+^ levels were monitored after the addition of the G_q_-agonist and analog of acetylcholine Oxo-M (10 μM). Comparison of neurons treated with PBS control or α-Syn fibrils revealed that addition of 10 μM Oxo-M resulted in a significantly larger increase in cytoplasmic Ca^2+^ from neurons exposed to α-Syn fibrils (Figure 5B), consistent with elevated PI(4,5)P_2_ increasing IP_3_-mediated Ca^2+^ release. This heightened Ca^2+^ release was confined to α-Syn fibrils, as two other pathogenic fibrils, Aβ and tau, failed to enhance G_q_-mediated responses (Figure S4A). Similar results were observed in HEK293T cells (Figure 5C) or HeLa cells (Figures S4B and S4C) overexpressing α-Syn^A53T^ and treated with the purinergic receptor G_q_-agonist UTP (10 μM), neurons treated with a Bradykinin receptor G_q_-agonist (100 μM; Figures S4D and S4E), and α-Syn^A53T^ patient fibroblasts treated with UTP (Figure S4F). In addition to IP_3_-mediated Ca^2+^ release, cytoplasmic Ca^2+^ levels were also elevated in cells treated with α-Syn^A53T^ (Figure S4G). Thus, α-Syn fibrils and α-Syn^A53T^ increase G_q_PCR-mediated Ca^2+^ release across multiple cell types. Underscoring a role for the PIP5K1γ enzyme in facilitating increases in Ca^2+^ release, treatment with the PIP5K1γ inhibitor UNC3230 normalized Ca^2+^ responses in cells overexpressing α-Syn^A53T^ back to a control range (Figures 5D and 5E), indicating that upstream increases in PI(4,5)P_2_ play an important role.

Following hydrolysis of PI(4,5)P_2_, soluble IP_3_ diffuses and binds to IP_3_Rs to release Ca^2+^. The most prevalent IP_3_R isoform in neurons is IP_3_R type 1 (IP_3_R1); therefore, to test if IP_3_R1 specifically mediates α-Syn-dependent increases in G_q_PCR Ca^2+^ release through IP_3_R1, we transfected *IP*_*3*_*R1*^−/−^ cells with α-Syn^A53T^ and discovered that loss of IP_3_R1 resulted in cells being refractory to expressional changes in α-Syn (Figures 5F and 5G). These data suggest that augmented PI(4,5)P_2_ levels increase G_q_PCR-mediated Ca^2+^ release specifically through IP_3_R1. This is aligned with published literature that immobile IP_3_R1s close to the PM at ER-PM junctions are preferentially licensed to release Ca^2+^.^76^

We have recently demonstrated that deviant increases in IP_3_R1 clustering are a key player in neuronal cell death in the neurodegenerative Niemann-Pick type C (NPC) disease.^73,77^ IP_3_R clustering is governed by several factors, including IP_3_ production. Clustering of IP_3_R leads to enhanced cooperativity and a higher propensity to release Ca^2+^, leading to oscillations in intracellular Ca^2+^ levels. To determine if enhanced PI(4,5)P_2_ drives IP_3_R1 clustering, we took several complementary fluorescent approaches. First, using a cell line that has endogenous IP_3_R1 tagged with GFP (GFP-IP_3_R1^76^), we found that overexpression of α-Syn^A53T^ significantly increased IP_3_R1 puncta intensity and area near the PM (Figures 5H and 5I). Similar results were observed in MAP2-postive hippocampal neurons transfected with α-Syn^A53T^ compared with control neurons (Figures 5J and 5K). To test if α-Syn-dependent increases in PIP5K1γ and/or PI(4,5)P_2_ were central drivers in enhanced IP_3_R1 clustering, we treated cortical neurons with α-Syn fibrils under conditions of UNC3230 (to inhibit PIP5K1γ production of PI(4,5)P_2_) and fixed and stained neurons for the dendritic marker MAP2 and for IP_3_R1. Quantification of IP_3_R1 clusters from MAP2-positive cells revealed that neurons treated with UNC3230 exhibited decreased IP_3_R1 immunostaining compared with control neurons, while neurons with α-Syn fibril treatment demonstrated increased total puncta area and integrated density (Figures 5L and 5M). Cotreatment of α-Syn fibrils with UNC3230 rescued IP_3_R1 total puncta area, and puncta integrated density back into a control range (Figures 5L and 5M). Collectively, these data present evidence that α-Syn-dependent increases in PI(4,5)P_2_ influence the distribution of IP_3_R1 clusters to enhance G_q_PCR-mediated Ca^2+^ release.

### α-Syn-dependent augmentation of IP_3_R clustering increases mitochondrial Ca^2+^, reactive oxygen species, and neuronal cytotoxicity

**Figure 6.**
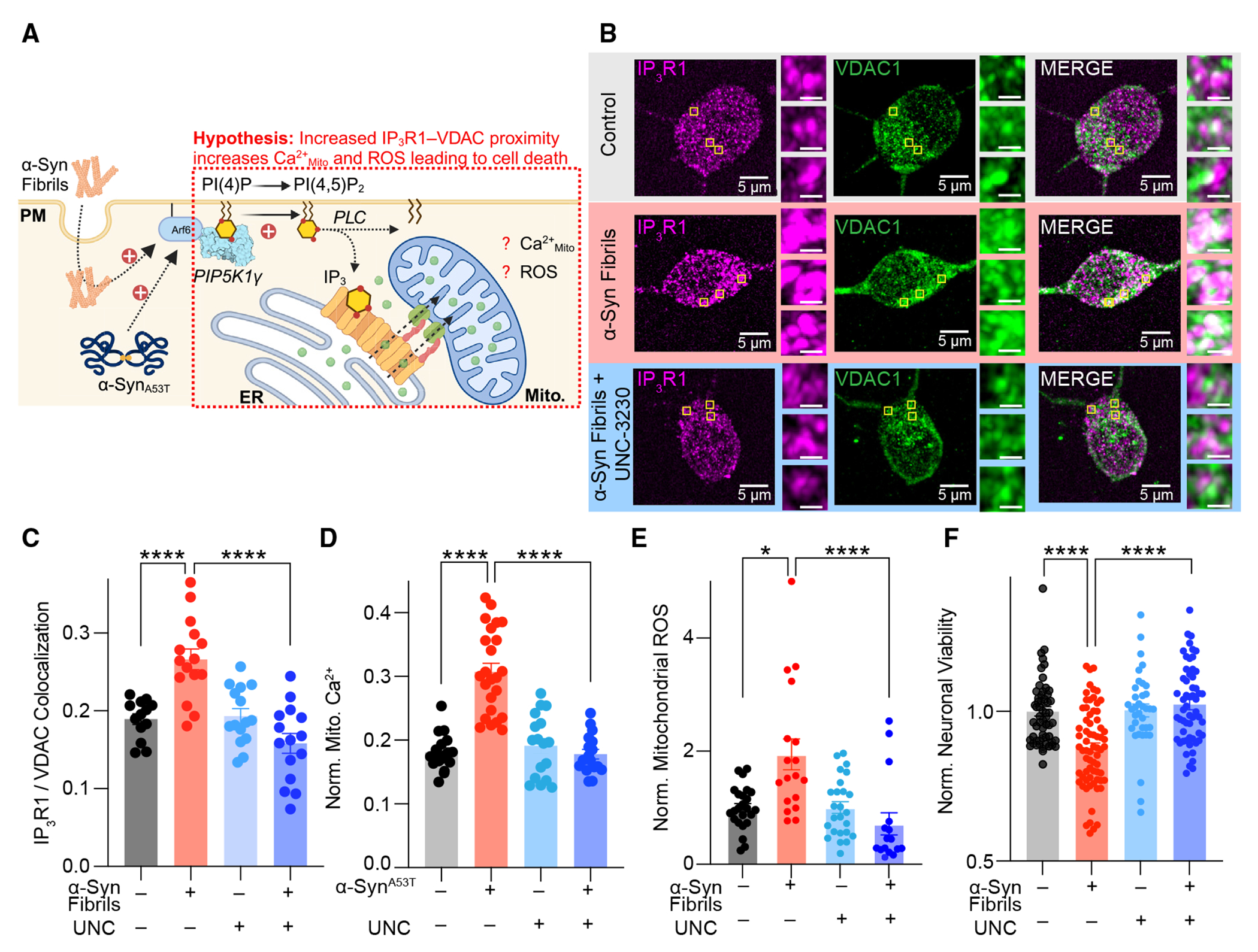
α-Syn-dependent augmentation of PI(4,5)P_2_ increases IP3R1-VDAC proximity, mitochondrial Ca^2+^, ROS, and neuronal cytotoxicity (A) Schematic of hypothesis to be tested: α-Syn-dependent increases in IP_3_R1-VDAC proximity potentiate mitochondrial Ca^2+^. (B) Representative confocal images of control and α-Syn fibril-treated neurons with or without treatment of UNC3230, stained for IP_3_R1 (magenta) and VDAC1 (green). (C) Quantification of the ratio of overlapping VDAC1/IP_3_R pixels divided by total IP_3_R1 pixels. (D) Quantification of normalized mitochondrial Ca^2+^ levels in control and α-Syn^A53T^-transfected HEK293T cells with or without treatment of 100 nM UNC3230. (E) Quantification of normalized mitochondrial ROS from H_2_DCFDA fluorescence in control and α-Syn fibril-treated mouse cortical neurons with or without treatment of 100 nM UNC3230. (F) Quantification of cell viability assay in control and α-Syn fibril-treated mouse cortical neurons with or without treatment of 100 nM UNC3230. Statistical analyses for (C)–(F) were two-way ANOVAs. Error bars represent the standard error of the mean. *p < 0.05; ****p < 0.0001.

Enhanced clustering of IP_3_R1 has been shown to positively enhance Ca^2+^ release.^73,78,79^ Furthermore, IP_3_R1s leak Ca^2+^ in response to spontaneous basal activity of PLC.^80,81^ At ER-mitochondrial (ER-Mito) interfaces, IP_3_R1s cluster opposite voltage-dependent anion channels (VDACs) to facilitate the transfer of Ca^2+^ from the ER to mitochondria to influence bioenergetics and maintain cellular homeostasis.^82^ Alterations in IP_3_R1-VDAC interactions have been demonstrated to cause dysfunctional Ca^2+^ transfer to Mito and have been proposed as a driver of neurodegeneration.^73^ Given the augmented IP_3_R1 clustering observed in neurons following α-Syn fibril treatment (Figure 5), we next asked if IP_3_R1-VDAC1 interactions and Ca^2+^_Mito_ are also enhanced, leading to cellular toxicity. To begin, we treated neurons with α-Syn fibrils and fixed and immunolabelled against IP_3_R1 and VDAC to map potential interactions between the proteins. Quantification of super-resolution images revealed that α-Syn fibrils increased the fraction of IP_3_R1 pixels colocalized with VDAC1 (Figures 6B and 6C). These data are aligned with previous reports of enhanced ER-Mito contacts with elevated α-Syn expression.^83^ Next, we expressed a genetically encoded Ca^2+^ indicator targeted to mitochondria (Mito-RCaMPh1^84^) to ask if Ca^2+^_Mito_ is altered by pathological α-Syn. Quantification of Mito-RCaMPh1 intensities between cells with or without α-Syn^A53T^ transfection revealed that α-Syn significantly increased Ca^2+^_Mito_ (Figure 6D), similar to what has been previously reported.^85^ To understand if alterations in Ca^2+^_Mito_ are accompanied by changes in cell health, we measured reactive oxygen species (ROS) and cell viability. Mitochondria are an important source of ROS, with ROS production linked to Mito damage in a range of pathologies, including neurodegeneration. Measurement of ROS in control, vehicle-treated neurons or neurons treated with α-Syn fibrils revealed a significant, 2-fold increase in Mito ROS levels (Figure 6E) that correlated with a decreased in neuronal viability (Figure 6F). To test for a role of the PIP5K1γ-PI(4,5)P_2_-IP_3_R1 signaling axis in mediating changes in Mito health, we treated neurons with the PIP5K1γ inhibitor UNC3230, which decreases α-Syn-dependent increases in PI(4,5)P_2_ (Figure 2), PIP5K1γ (Figures 2 and 3), and IP_3_R1 (Figure 5), and found that concurrent treatment of α-Syn fibrils with UNC-3230 normalized IP_3_R1-VDAC colocalization (Figure 6B), Ca^2+^_Mito_ (Figure 6D), Mito ROS (Figure 6E), and cell viability (Figure 6F) back to control levels. Taken together, these data suggest that α-Syn-dependent increases in PI(4,5) P_2_ initiate a damaging feedforward signaling cascade that reorganizes the nanoscale distribution of IP_3_R1 to alter intracellular Ca^2+^ signaling networks to perturb Mito function and trigger neurotoxicity.

## DISCUSSION

α-Syn has been reported to play a vital role in the progression of many pathologies through its ability to form the building block of Lewy bodies, the deviant protein deposits that accumulate, deposit, and propagate between brain regions during different dementias, including Lewy body dementia, PD, and Alzheimer’s disease. Despite these strong correlations, the molecular connection(s) that link α-Syn accumulation and neurodegeneration remain unknown. Here, we provide evidence that the membrane lipid PI(4,5)P_2_, a critical organizer of membrane events, is increased across several models of altered α-Syn, including different brain neurons affected in Lewy body dementias. The consequences of elevated cellular PI(4,5)P_2_ levels are enhanced α-Syn aggregation and augmented IP_3_-mediated Ca^2+^ release leading to Mito dysfunction. Given the importance of PI(4,5)P_2_ for orchestrating membrane events in neurons,^86^ we expect other signaling reactions/cascades to be perturbed as well. The molecular events connecting α-Syn and PI(4,5)P_2_ appear to involve ARF6-dependent recruitment of the PI(4,5)P_2_-metabolizing enzyme, PIP5K1γ, as selectively targeting ARF6 protein abundance or activity rescues cellular phenotypes. These data position ARF6-PIP5K1γ as not only important regulators of PM identity in health but also as key targets to potentially uncouple the destructive downstream consequences of α-Syn accumulation in synucleinopathies (for model, see Figure S5).

α-Syn is an intrinsically disordered protein that exists in a variety of conformations, with the prevailing, but not universal,^29,87,88^ hypothesis positing that its cumulative oligomerization at membranes correlates with toxicity.^30,89,90^ An important mediator that facilitates the binding of α-Syn to membranes are lipids with positive charges in the N terminus of α-Syn electrostatically interacting with negatively charged lipid phosphate head groups.^89^ Interestingly, the lipid sensing regions of α-Syn and the genetic mutations that facilitate its pathological aggregation occur at the same domain, presenting a model were the local lipid environment could seed and nucleate the aggregation of α-Syn. Physiologically, stabilization of α-Syn by lipids promotes interactions with SNARE complex proteins^2,6,91^ to influence synaptic vesicle endocytosis,^4,92^ while pathophysiologically, lipid-protein interactions increase the propensity of α-Syn membrane aggregation.^93,94^ In the present study, we demonstrate that α-Syn fibrils or disease mutations increase PM lipid PI(4,5)P_2_, which is in agreement with reports from other groups regarding an important role for this lipid in PD.^34,39,40,95^ Given the importance of α-Syn for synaptic vesicle endocytosis and exocytosis, both PI(4,5)P_2_-dependent events^96^ that facilitate prion-like spread of Lewy bodies^42,92,97^ and the ability of PI(4,5)P_2_ to bind and aggregate α-Syn^32,33,35^ indicate that PI(4,5)P_2_ may play an important role in the progression and spread of α-Syn pathology. Supporting this hypothesis, data herein demonstrate that inhibition of PIP5K1γ, which is critical for mediating α-Syn-dependent increases in PI(4,5)P_2_, normalizes α-Syn aggregates and rescues neurotoxic events. Thus, a picture is developing where the appropriate PI(4,5)P_2_-to-α-Syn ratio is required for membrane targeting and orchestration of neuronal function; however, as this delicate balance shifts, such as during aging^98^ or synucleinopathies, it may lead to a local membrane environment that favors aggregation and supports spreading neurotoxicity.

In the present study, we propose that both α-Syn fibrils and α-Syn^A53T^ disease mutation mediate increases in PI(4,5)P_2_ through increased expression and localization of the phosphoinositide-metabolizing kinase PIP5K1γ, a key regulator of synaptic vesicle trafficking.^38^ Further underscoring a connection between α-Syn and PIP5K1γ, inhibiting PIP5K1γ catalytic activity rescues PM PI(4,5)P_2_ and cellular phenotypes. We establish that PIP5K1γ recruitment appears to be under the control of ARF6 since (1) ARF6 distribution tracks that of PIP5K1γ, (2) expression of dnARF6 rescues α-Syn-dependent elevations in PIP5K1γ, and (3) *ARF6*^−/−^ neurons no longer exhibit elevations in PI(4,5)P_2_ or aggregation of α-Syn at the PM. What links α-Syn fibrils/α-Syn^A53T^ to ARF6-dependent recruitment of PIP5K1γ remains to be seen, although given that ARF6 localization is controlled by its GTP/GDP status, this strongly suggests that ARF6 GEF (guanine nucleotide exchange factors)/GAPs (GTPase activating proteins) or scaffolding effectors may be immediate upstream players linking α-Syn to ARF6 function. Supporting the hypothesis that homeostatic control of ARF6-GEF/GAP signaling is critical for neuronal health, ARF6 and its GEFs are essential for the maintenance of dendritic spines^99^ and axonal branching and elongation,^100^ while their dysfunction has been implicated in neurological disorders including fragile X syndrome^101^ and intellectual disability.^102^ Finally, the critical observation that ARF6 expression correlates with Alzheimer’s disease progression^103^ recommends more targeted clinical research to determine if ARF6 and its effectors represent potential targets to slow neurodegenerative disease progression.

One of the hallmarks of dopaminergic neurons of the substantia nigra is their vulnerability to sustained elevations in cytosolic Ca^2+^. Their poor endogenous Ca^2+^ buffering capability^104^ and autonomous oscillations in intracellular Ca^2+^ concentrations^105^ render them vulnerable to stressors that further elevate intracellular Ca^2+^ levels. Our data contribute to this list of vulnerable Ca^2+^ features observed in neurons with altered α-Syn expression. We find that a downstream consequence of elevated PI(4,5)P_2_ is enhanced IP_3_R-mediated Ca^2+^ release, which reorganizes IP_3_R1 in ER membranes. We determine that enhanced clustering of IP_3_R1, a driver of neuronal death in the neurodegenerative NPC disease,^73^ correlates with potentiation of Ca^2+^_Mito_, ROS, and neuronal toxicity. α-Syn reorganization of IP_3_R1 distribution seems to be dependent on IP_3_ generation, as inhibiting PI(4,5)P_2_ production rescues the nanoscale organization of IP_3_R1 and abrogates neurotoxic phenotypes. These data align well with recent findings that inhibition of the inositol-1,4,5-triphosphate kinase B (ITPKB), which increases cytoplasmic IP_3_ concentrations, potentiates α-Syn pathology.^106^ Importantly, we show for the first time that targeting upstream elevations in PI(4,5)P_2_ levels in models of α-Syn pathology rescues ER-Mito membrane contact site integrity and Mito Ca^2+^ handling, demonstrating that PIP5K1γ plays a crucial role in determining Mito-dependent α-Syn pathophysiology.

In summary, we present evidence that α-Syn fibrils or disease mutations drive increases in the abundance of PM PI(4,5)P_2_, leading to enhanced α-Syn aggregation and aberrant IP_3_ Ca^2+^ signaling, which have deleterious consequences for neuronal health. Given the diverse range of cellular pathways that phosphoinositides integrate and control, their role in other neurodegenerative diseases, and our finding that PI(4,5)P_2_ is a critical rheostat for the development of cellular α-Syn pathophysiology, invites further investigations to elucidate how careful modulation of PI(4,5)P_2_ abundance and distribution could be exploited in the clinic to slow Lewy body-related dementias.

### Limitations of the study

Within the context of this investigation, we have unveiled the noteworthy correlation that both exogenous treatment of α-Syn fibrils and cytoplasmic expression of α-Syn^A53T^ influence PI(4,5)P_2_ homeostasis and Ca^2+^ signaling pathways. The molecular mechanism through which α-Syn fibrils and α-Syn^A53T^ mediate these identical effects are unknown. There is evidence that exogenous treatment of α-Syn fibrils can bind and permeabilize neuronal membranes to release α-Syn oligomers into the cytoplasm.^107^ Release of α-Syn oligomers may then act through a similar mechanism to aggregated α-Syn^A53T^, but more experiments explicitly designed to test this hypothesis are warranted. Moreover, the mechanistic pathways linking α-Syn fibrils/α-Syn^A53T^ and the heightened activity and localization of ARF6 at the PM remain unknown. Understanding the precise signaling connections between α-Syn fibrils, α-Syn^A53T^, and ARF6 that underlie enhancement of PI(4,5)P_2_ levels will be imperative to develop a full understanding of α-Syn aggregation.

## STAR★METHODS

### RESOURCE AVAILABILITY

#### Lead contact

All additional information and requests for resources, reagents, and methods should be directed to the lead contact, Eamonn J. Dickson (ejdickson@ucdavis.edu).

#### Materials availability

This study did not generate new reagents.

#### Data and code availability

All data reported in this paper will be shared by the lead contact upon request. This paper does not report original code. Any additional information required to reanalyze the data reported in this paper is available from the lead contact upon request.

### EXPERIMENTAL MODEL AND STUDY PARTICIPANT DETAILS

#### Animals and cell culture

All experiments involving animals were performed in accordance with protocols approved by the University of California Davis Animal Care and Use Committee (protocol #: 22644). For all datasets using isolated neurons, cultures were prepared from 6 to 8 male and female E15-18 pups. Embryonic hippocampal, cortical, and substantia nigra neurons were isolated from C57BL/6J (The Jackson Laboratory) or ARF6^−/−^ mice at embryonic day 15–18 (E15-18) of gestation and plated on 35 mm glass coverslips. To generate ARF6 conditional knockout mice, we obtained Arf6 floxed mice^109^ (Arf6tm1.1Gdp, The Jackson Laboratory #28669), and crossed them with Emx1-CRE mice (Emx1tm1(cre)Krj, The Jackson Laboratory #005628). WT and ARF6^−/−^ neurons (Emx1-CRE; Arf6 fl/fl) were cultured in media containing Neurobasal (21103-049; Gibco) supplemented with B27 (17504-044; Gibco), Glutamax (35050-061; Gibco), and 0.2% penicillin/streptomycin. 50% neuronal media was exchanged every 3 days for fresh media. WT (C57BL/6J) and α-Syn^A53T^ (Prnp-SNCA*A53T)83VleStrain, The Jackson Laboratory (JAX#:004479) brains were used for UHPLC-ms/ms measurements.

CHO, HEK293T, HeLa, and HEK293-Cas9-RFP cells (CRL-1573Cas9; ATCC) were cultured in DMEM (11995-065; Gibco) containing 10% FBS and 0.2% penicillin/streptomycin and passaged twice weekly at 1:20. Fibroblast cell lines from a healthy male patient (GM05659), a male patient with PD (AG20445), a healthy female patient (ND36091) and a female PD patient harboring α-Syn_A53T_ (NDS00188) with were acquired from NINDS human cell repository and the Coriell Institute. Fibroblasts were passaged twice weekly and were cultured in MEME (M5650; Sigma) containing 15% FBS, 2 mM L-glutamine, and 0.2% penicillin/streptomycin. eGFP-IP_3_R HeLa cells were a gift from Colin Taylor^76^ and were cultured in the same media conditions as wild-type HeLa cells. IP_3_R type-1^−/−^ HEK293 cells were purchased from Kerafast and were cultured in the same media as HEK293T cells. Doxycycline-inducible α-Syn-expressing SH-SY5Y cells were a gift from Muralidhar Hegde.^66^ Undifferentiated cells were treated with 3 μg/mL doxycycline hyclate (J60579; Alfa Aesar) for 72 h to induce α-Syn overexpression. All cell lines were incubated in 5% CO_2_ at 37°C.

### METHOD DETAILS

#### Transfections, plasmids, and siRNA

Lipofectamine 2000 (11668-019; Invitrogen), LTX (15338-030; Invitrogen), and RNAiMax (13778-030; Invitrogen) were used for 24-h cDNA transfections as per manufacturer’s recommendations for cultured cells. Neurons were transfected between DIV 5–8 and culture media was replaced with a 2:1 ratio of old:fresh media following neuronal transfections. Neuronal media was exchanged once per week while cultured. The following cDNA plasmids were used in the present study: PH_PLCδ1_ –CFP (gift from Tamas Balla), α-Syn^A53T^-GFP (gift from Bjoern Falkenberger^108^), GFP-PIP5KIgamma (Addgene: 22299^38^), pCAG mito-RCaMP1h (Addgene: 105013^84^), p3E-ARF6-DN (Addgene; 109592^110^). DsiRNA Duplex targeting PIP5K1γ and sgRNA targeting SNCA were purchased from Integrated DNA Technologies and transfected as per manufacturer’s recommendations.

#### Live cell airyScan super resolution imaging

Coverslips containing transfected cells were imaged in 2 mM Ca^2+^ Ringer’s solution (160 mM NaCl, 2 mM CaCl_2_, 1 mM MgCl_2_, 2.5 mM KCl, 10 mM HEPES, and 8 mM glucose) and were excited using 405 nm, 488 nm, or 594 nm lasers. Resulting light was collected using a Plan-Apochromat 63×/1.40 oil-immersion lens and a Zeiss 880 Airyscan microscope at room temperature (21°C). Images were processed with Airyscan post-image processing using Zen software. For cells transfected with PH-CFP and GFP-PIP5KIγ, mean intensity values of in-focus plasma membrane was divided by the mean intensity value of in-focus cytoplasm to obtain PM/Cyto ratio.

#### Lipid mass spectrometry and lipid kinase assay

Phosphoinositides were quantified as described previously.^47^ Briefly, an n-butanol and chloroform lipid extraction was performed on (i) neurons isolated from E18 animals and cultured for 14 days with PBS (control) or α-Syn fibrils (14 days) or (ii) 12 month old α-Syn^A53T^ murine brains (JAX strain number: 004479).^24^ After derivatization, samples were ran on a C4 column using an acetonitrile/formic acid gradient. Post-column eluate was infused with sodium formate and monitored using a Waters XEVO TQ-S MS/MS in multiple reaction monitoring (MRM), positive ion mode. Elution profiles were quantified by integrating area under peaks using MassLynx software. Peak areas of individual phosphoinositide species from the biological sample were normalized to synthetic standards and corrected for tissue amount using total protein. For measurement of PIP5K activity, a PI(4)P 5-kinase activity assay was used (Echelon; K-5700) according to manufacture instructions. Briefly, this is an ATP depletion assay which quantifies the remaining ATP levels in solution following addition of lysates from control (PBS only), α-Syn fibrils, or α-Syn^A53T^ treated/expressing neurons, DIV14.

#### Immunocytochemistry

Cultured cells were initially washed with PBS and fixed in 4% PFA for 10 min. Neurons were fixed between DIV 7–14. Cells were subsequently washed again then blocked with 20% Sea Block Blocking Buffer (37527; Thermo Scientific) containing 0.1% Triton X-100 (T8787; Sigma) for 1 h at 21°C. Cells were stained at 10 μg/mL overnight at 4°C with the following primary antibodies: anti-PIP5K1γ (gift from Dr. Pietro DiCamilli,^38^; anti-β-actin (MA1-91399; Invitrogen), anti-GAPDH (10494-1-AP; Proteintech), anti-α-synuclein [LB509] (ab27766; Abcam), anti-IP_3_R1 (75-035; Antibodies Inc.), anti-MAP2 (AB5622; Millipore), anti-MAP2 (ab11267; Abcam), anti-Ser(P)-129-α-synuclein (ab51253; Abcam), anti-VDAC1 (ab14734; Abcam). Cells were washed with PBS and incubated at 21°C for 1 h with the following secondary antibodies at 1:1,000 in blocking solution: Alexa Fluor 647 goat anti-mouse (A21236; Invitrogen), Alexa Fluor 555 goat anti-mouse (A21424; Invitrogen), Alexa Fluor 647 goat anti-rabbit (A21245; Invitrogen), Alexa Fluor 555 goat anti-rabbit (A21429; Invitrogen). z stack images were collected using a Plan-Apochromat 63×/1.40 oil-immersion lens and a Zeiss 880 Airyscan microscope at room temperature (21°C). Images were processed with Airyscan post-image processing using Zen software. Z-stacks were converted to a singular maximum intensity projection image in ImageJ. Analysis parameters Subtract Background, Median Filter, Threshold, and Particle Analysis were held constant for all images in ImageJ.

#### Single molecular localization microcopy

Undifferentiated SH-SY5Y cells, seeded on coverslips (No. 1.5) were fixed in 4% PFA for 10 min, blocked with 20% SeaBlock containing 0.1% Triton X-100 for 1 h at 21°C, and stained with anti-α-synuclein (ab27766; Abcam) overnight at 4°C. Cortical neurons were treated with α-Syn fibrils or vehicle control for between 72 h and 14 days,^23^ fixed and stained for anti-PIP5K1γ (gift from Dr. Pietro DiCamilli^38^). Cells were incubated for 1 h in Alexa Fluor 647 donkey anti-rabbit (A31573; 1:1,000; Invitrogen) or Alexa Fluor 647 goat anti-mouse (A21236; 1:1000; Invitrogen) secondary antibody in blocking solution. Coverslips were then mounted onto glass depression slides (neoLab, Heidelberg, Germany) with a cysteamine (MEA)-catalase/glucose/glucose oxidase (GLOX) imaging buffer containing TN buffer (50 mM Tris pH 8.0, 10 mM NaCl), a GLOX oxygen scavenging system (0.56 mg mL– 1 glucose oxidase, 34 μg mL–1 catalase, 10% w/v glucose) and 100 mM MEA. Twinsil dental glue (Picodent, Wipperfürth, Germany) and aluminum tape (T205–1.0 - AT205; Thorlabs Inc., Newton, NJ, USA) were used to hold the coverslip in place on the slide and to exclude oxygen. Images were captured using a Leica Infinity TIRF super-resolution microscope equipped with a 163×1.49 NA TIRF oil immersion objective and a Hamamatsu orca flash 4.0 camera. Particle Analysis in ImageJ was conducted using 20 nm pixel size.

#### Protein extraction and western blot

Protein from cultured fibroblasts was harvested and lysates were blotted as previously described.^111^ anti-PIP5K1γ (ABS190; 1:300; Sigma) and anti-β-actin (MA1-91399; 1:1000; Thermo-Fisher) were applied to transferred membranes overnight at 4°C. Blot bands were detected by Sapphire Biomolecular Imager (Azure Biosystems) after 1 h incubation in the following secondary antibodies: goat anti-rabbit 680RD (P/N 926–68071, 1:10,000; LI-COR), goat anti-Mouse 800CW (P/N 925–32210, 1:10,000; LI-COR). Images were processed on ImageJ using the BioImporter plugin tool to calculate the protein expression for each band. Protein abundance was first normalized to beta-actin intensity then normalized to control cell intensity.

#### Ca^2+^ imaging

Cells were incubated in 2 mM Ca^2+^ Ringer’s solution containing 5 μM Fluo-4 AM (F14201; Invitrogen) and 0.1% pluronic acid (P3000MP; Invitrogen) to permeabilize cells at 21°C for 30 min, followed by deesterification in Fluo-4-free Ringer’s solution for 30 min. Cells were bathed in 2 mM Ca^2+^ Ringer’s solution and excited by a 488 nm laser and the resulting fluorescence was monitored using an inverted microscope with a Plan-Apochromat 40×/1.40 oil objective, connected to an Andor W1 spinning-disk confocal with a Photometrics Prime 95B camera. Image acquisition occurred at 21°C every 5 s using Micromanager software. At 100 s, cells were perfused with 100 μM UTP or bradykinin in 2 mM Ca^2+^ Ringer’s solution for 100 s. At 200 s, cells were perfused with 2 mM Ca^2+^ Ringer’s solution without G_q_ agonist and imaged until 400 s. Images stacks were analyzed in ImageJ. An ROI was drawn in the cytosol of Fluo-4-loaded cells and measured for fluorescence. Intensity was normalized to minimum intensity value before G_q_ agonist application. Amplitude and area under the curve measurements were made by GraphPad Prism.

#### IP_3_R-VDAC1 colocalization assay

Mouse cortical neuron cultures were treated with or without α-Syn fibrils 72 h prior to fixation and/or treated with UNC-3220 24 h prior to fixation. Cultured cells were fixed, blocked, and stained as described in immunocytochemistry. Coverslips were excited by a 488 nm or 633 nm laser, and resulting light was collected using a Plan-Apochromat 63×/1.40 oil-immersion lens and a Zeiss 880 Airyscan microscope at room temperature (21°C). z stack Images were processed with Airyscan post-image processing using Zen software. Z-stacks were converted to a singular maximum intensity projection image in ImageJ. Analysis parameters Subtract Background, Median Filter, and Threshold were held constant for all images in ImageJ. Threshold images of each channel were converted to binary images and the resulting IP_3_R channel was multiplied by the VDAC1. IP_3_R-positive and VDAC1-positive (overlapping) pixels were divided by total number of IP_3_R-positive pixels to obtain percent colocalization.

#### Mitochondrial Ca^2+^ assay

HEK293T cells with or without α-Syn_A53T_ overexpression were transfected with Mito-RCamPh1. UNC-3230 and vehicle treatment groups were treated 24 h prior to imaging. Cells were incubated in 2 mM Ca^2+^ Ringer’s solution and excited by a 546 nm laser. Resulting light was collected using a Plan-Apochromat 63×/1.40 oil-immersion lens and a Zeiss 880 Airyscan microscope at room temperature (21°C). Images were processed with Airyscan post-image processing using Zen software. After initial image capture, cells were perfused with 20 mM Ca^2+^ Ringer’s solution containing 2.5 μM ionomycin for 5 min at 21°C. Cells were excited by a 546 nm laser for a second time. Pre-inomycin and post-ionomycin images were compared for each treatment group. A threshold was applied to images in a consistent manner to obtain mitochondrial ROIs for RCaMPh1 fluorescence. The intensity ratio of post-inomycin/pre-ionomycin of mito-RCaMPh1 was taken for each cell, with higher ratios indicating less mitochondrial Ca^2+^.

#### ROS assay

Mouse cortical neuron cultures were treated with or without α-Syn fibrils 72 h prior to imaging and/or UNC-3220 24 h prior to imaging. Coverslips were incubated in 2 mM Ca^2+^ Ringer’s solution containing 2′,7′-dichlorodihydrofluorescein diacetate (H_2_DCFDA; D399; Invitrogen) for 20 min at room temperature (21°C). Upon cleavage of acetate groups by ROS, non-fluorescent H_2_DCFDA is converted to fluorescent 2′,7′-dichlorofluorescein (DCF). Cells were excited with a 488 nm laser. Resulting light was collected using a Plan-Apochromat 63×/1.40 oil-immersion lens and a Zeiss 880 Airyscan microscope at room temperature (21°C). Images were processed with Airyscan post-image processing using Zen software. ROIs were drawn around resulting mitochondrial fluorescence and intensity was recorded for each cell. Intensity values for each group were normalized to control values.

#### Cell viability assay

Cell viability assay was conducted as per manufacturer’s recommendations (K502-100; BioVision). Live DIV 13 cortical neurons were washed once with 2 mM Ca^2+^ Ringer’s solution and loaded with 1 mL assay buffer containing 2 μL Live cell staining dye and 1 μL Dead cell staining dye. Cells were immediately excited using a 488 nm and 564 nm LED. Single-plane images were collected using a Plan-Apochromat 63μ/1.40 oil-immersion lens and a Zeiss 880 Airyscan microscope at room temperature (21°C). Images were analyzed using ImageJ. Ratio of green (live) to red (dead) fluorescence was taken for each neuron.

#### Reagents

Doxycycline (J60579; Alfa Aesar) was dissolved in diH_2_O and SH-SY5Y cells were treated at 3 μg/mL for 72 h. UNC-3230 (5271; Tocris) and ISA-2011B (HY-16937; MedChemExpress) were dissolved in DMSO and cells were treated at 100 nM for 24 h prior to transient transfection or 24 h prior to fixation. Bradykinin acetate salt (B3259; Sigma-Aldrich) was dissolved in diH_2_O and perfused at 100 μM in 2 mM Ca^2+^ Ringer’s Solution. Oxotremorine M (O100; Sigma-Aldrich) was dissolved in diH_2_O and perfused at 100 μM in 2 mM Ca^2+^ Ringer’s Solution. UTP trisodium salt (U6625; Sigma-Aldrich) was dissolved in diH_2_O and perfused at 100 μM in 2 mM Ca^2+^ Ringer’s Solution. Active type 1 recombinant human α-Syn pre-formed fibrils (SPR-322; StressMarq Biosciences) were dissolved in PBS and sonicated for 10 min prior to treatment at 4 μg/mL for 72 h– 14 days. Irrespective of length of α-Syn fibril treatment, all isolated neuronal cultures spent a total of 14 days in culture from initial addition of α-Syn fibrils. α-Syn fibrils were confirmed by immunofluorescence following Triton X- permeabilization and Thioflavin S staining.^23^

### QUANTIFICATION AND STATISTICAL ANALYSIS

#### Data analysis and figure preparation

Microsoft Excel, and GraphPad Prism were used to analyze all data. ImageJ was used to process and analyze images. Diagrams were made using BioRender. All datasets were repeated a minimum of 3 times. Data are presented as mean +SEM For datasets with two treatment groups, parametric Student’s *t* tests were conducted to determine significance. For datasets with more than two treatment groups, parametric one-way ANOVA tests were conducted, and significance was determined by comparing mean values of each group from both Normality tests were conducted on all treatment groups, with groups not passing subject to nonparametric tests. In Figures 1, 2, 4, and 5 certain datasets are presented using normalized values with each data group scaled to the control group which we set to 1. p values <0.05 were considered to be statistically significant.

## Supplementary Material

Supplemental information

## Figures and Tables

**Table:** KEY RESOURCES TABLE

REAGENT or RESOURCE	SOURCE	IDENTIFIER
Antibodies		
anti-PIP5K1コ	Gift from Pietro De Camilli	Di Paolo et al.^38^
Anti-Synaptojanin-1	Invitrogen	RRID:AB_2201023
anti-PIP5K1α	Proteintech	RRID:AB_2164696
anti-β-actin	Invitrogen	A1-91399
Anti-GAPDH	Proteintech	RRID:AB_2263076
anti-α-synuclein [LB509]	Abcam	RRID:AB_727020
anti-IP3R1	Antibodies Inc.	RRID:AB_10000362
anti-MAP2	Millipore	RRID:AB_91939
anti-Ser(P)-129-α-synuclein	Abcam	RRID:AB_869973
anti-VDAC1	Abcam	RRID:AB_443084
Alexa Fluor 647 goat anti-mouse	Invitrogen	RRID:AB_2535805
Alexa Fluor 555 goat anti-mouse	Invitrogen	RRID:AB_141780
Alexa Fluor 647 goat anti-rabbit	Invitrogen	RRID:AB_141775
Alexa Fluor 555 goat anti-rabbit	Invitrogen	RRID:AB_2535850
Alexa Fluor 647 donkey anti-rabbit	Invitrogen	RRID:AB_2536183
goat anti-rabbit 680RD	LI-COR	RRID:AB_10956166
goat anti-Mouse 800CW	LI-COR	RRID:AB_2687825
Chemicals, peptides, and recombinant proteins		
Neurobasal	Gibco	21103–049
B27	Gibco	17504–044
Glutamax	Gibco	35050–061
DMEM	Gibco	11995–065
MEME	Sigma	M5650
Doxycycline hyclate	Alfa Aesar	J60579
Lipofectamine 2000	Invitrogen	11668–019
LTX	Invitrogen	15338–030
RNAiMax	Invitrogen	13778–030
Sea Block Blocking Buffer	Thermo Scientific	37527
Triton X-100	Sigma	T8787
Fluo-4 AM	Invitrogen	F14201
Pluronic acid	Invitrogen	P3000MP
UNC-3220	Tocris	52713
2′,7′-dichlorodihydrofluorescein diacetate	Invitrogen	D399
Bradykinin acetate	Sigma-Aldrich	B3259
Oxotremorine M	Sigma-Aldrich	O100
UTP trisodium salt	Sigma-Aldrich	U6625
Human α-Syn pre-formed fibrils	StressMarq Biosciences	SPR-322
Thioflavin S	Sigma-Aldrich	T1892
Human Tau-441 (2N4R) Wild-Type Preformed Fibrils	StressMarq Biosciences	SPR-480
Human Synthetic Amyloid Beta 1–42 Preformed Fibrils	StressMarq Biosciences	SPR-487
Critical commercial assays		
PI(4)P 5-kinase activity assay	Echelon	K-5700
Cell Viability assay	BioVision	K502-100
ISA-2011B	MedChemExpress	HY-16937
Experimental models: Cell lines		
HEK293T	Sigma	96121229-1VL
HeLa	ATCC	CCL-2^™^
CHO	ATCC	CRL-11268
HEK293-Cas9-RFP cells	ATCC	CRL-1573Cas9
Fibroblast (Male; WT)	Coriell	GM05659
Fibroblast (Male; PD)	Coriell	AG20445
Fibroblast (Female; WT)	Coriell	ND36091
Fibroblast (Female; PD)	Coriell	NDS00188
eGFP-IP_3_R HeLa cells	Collin Taylor (Cambridge)	Thillaiappan et al.^76^
IP_3_R type-1 ^−/−^	Kerafast	EUR034
SH-SY5Y Cells	Muralidhar Hegde	Vasquez et al.^66^
Experimental models: Organisms/strains		
C57BL/6J	JAX	000664
α-Syn^A53T^ (Prnp-SNCA*A53T)	JAX	004479
Arf6tm1.1Gdp	JAX	28669
Emx1tm1(cre)Krj	JAX	005628
Recombinant DNA		
PH^pLCδ1^ –CFP	Tamas Balla	N/A
α-Syn^A53T^-GFP	Bjoern Falkenberger;	Opazo et al.^108^
GFP-PIP5KIgamma	Addgene	RRID:Addgene_22299
pCAG mito-RCaMP1h	Addgene	RRID:Addgene_105013
p3E-ARF6-DN	Addgene	RRID:Addgene_109592
LYN11-FRB-CFP	Addgene	RRID:Addgene_38003
PJ-5P	Addgene	RRID:Addgene_3800
PJ-Dead	Addgene	RRID:Addgene_38002
PH-Btk-GFP	Addgene	RRID:Addgene_51463
pEGFP-2xFYVE	Addgene	RRID:Addgene_140047
Software and algorithms		
Zen software	Zeiss	N/A
Microsoft Excel	Microsoft	N/A
Prism	GraphPad	N/A
BioRender		N/A

## Highlights

- α-Syn fibrils facilitate ARF6-PIP5K1γ increases in PI(4,5)P_2_
- α-Syn-driven increases in PI(4,5)P_2_ represent a key proximal step in α-Syn aggregation
- α-Syn elevations in PI(4,5)P_2_ increase mitochondrial Ca^2+^ to drive neuronal cytotoxicity
