# Supplementary material for: α-Synuclein-dependent increases in PIP5K1γ drive inositol signaling to promote neurotoxicity
Source: Cell Rep. Author manuscript; Available in PMC 2024 Apr 12. (PMC11010634; doi:10.1016/j.celrep.2023.113244)
Supplement: Supplemental information [file NIHMS1980419-supplement-Supplemental_information.pdf]

Cell Reports, Volume *42*

Supplemental information

α-Synuclein-dependent increases in PIP5K1γ drive inositol signaling to promote neurotoxicity

Jonathan D. Horvath, Maria Casas, Candice Kutchukian, Sara Creus Sánchez, Melissa R. Pergande, Stephanie M. Cologna, Sergi Simó, Rose E. Dixon, and Eamonn J. Dickson

Supplemental Figures.

Figure S1. α-Synuclein fibrils and disease mutations increase aggregation and PM $PI(4,5)P_2$ across multiple cell types.

Figure S2. α-Syn-dependent increases in PM $PI(4,5)P_2$ does not appear to involve PIP5K1α, synaptojanin 1, or increased recruitment by NIR2.

Figure S3. Knocking down PIP5K1γ decreases α-Syn aggregation.

Figure S4. α-Syn fibrils and α-Syn^{A53T} augment IP_3-mediated Ca^{2+} release.

Figure S5. Model of how α-Syn fibrils and a-SynA53T mediate increases in plasma membrane PI(4,5)P2 to drive α-Syn aggregation and toxic elevations in mitochondrial Ca^{2+}.

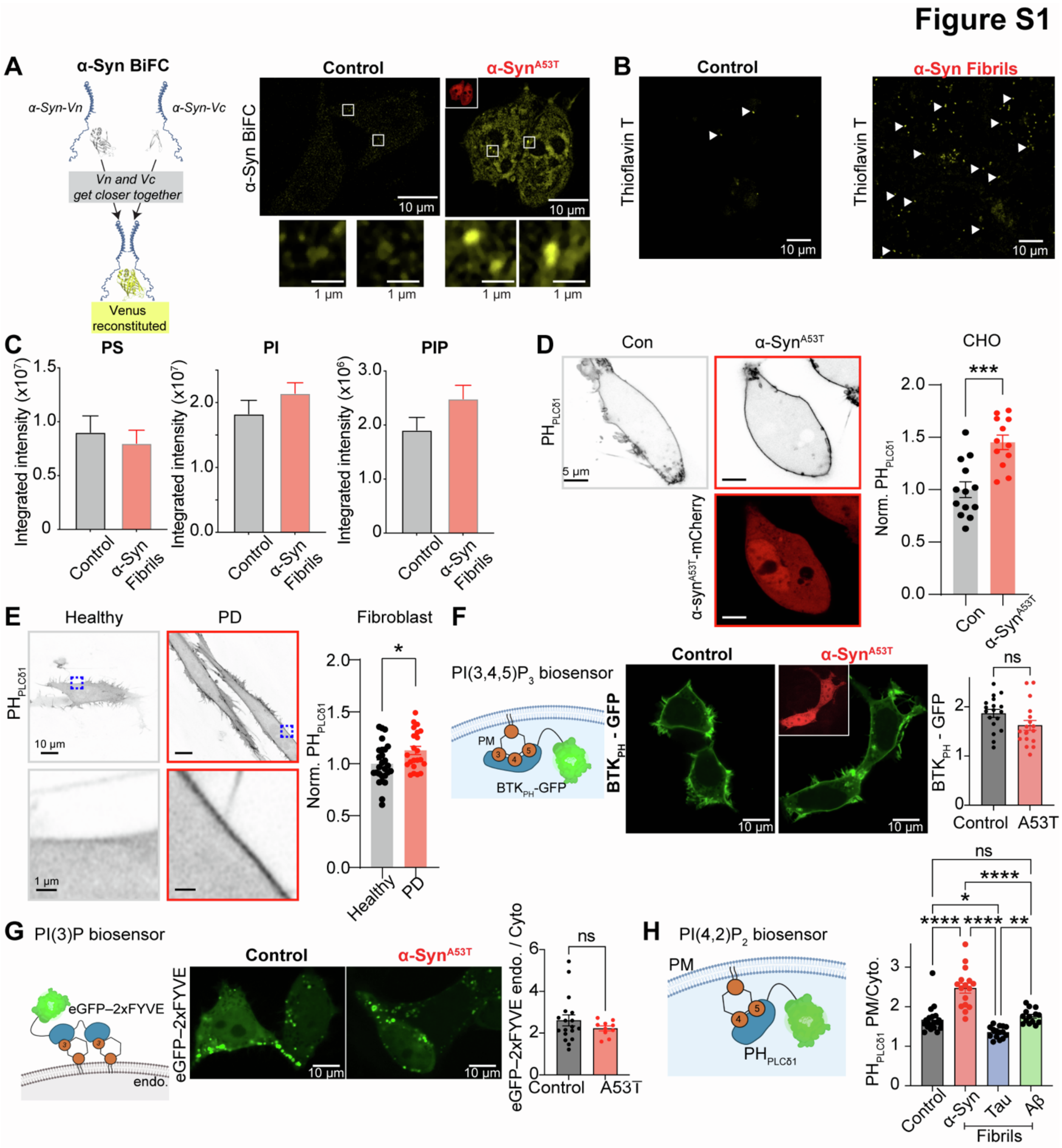

Figure S1. α-Synuclein fibrils and disease mutations increase aggregation and PM PI(4,5)P₂ across multiple cell types. (A) Left: schematic of experiment and how BiFC technic works. Right: Cortical neurons expressing V_n and V_c α-Syn with or without α-Syn^{A53T}. Note that while Venus puncta do not allow discrimination between amyloid and amorphous aggregation, these Venus puncta observed with Syn^{A53T} expression are similar in size to Thioflavin T puncta following incubation with α-Syn fibrils (see Figure

S1B) and Syn^{A53T} expression[1,2]. **(B)** Isolated cortical neurons treated with PBS or α-Syn fibrils for 14 days before being fixed and stained for protein aggregates (yellow) using Thioflavin T. **(C)** Mass spec lipid analysis of phosphatidylserine, phosphatidylinositol, and phosphatidylinositol 4-phosphate from neurons treated with PBS control of α-Syn fibrils. **(D)** Representative confocal images of control and α-Syn^{A53T}-transfected Chinese Hamster Ovary (CHO) cells transfected with $PH_{PLC\delta1}$-CFP. Quantification of PM/cytoplasm intensity ratio of $PH_{PLC\delta1}$ in CHO cells. α-Syn group values are normalized to control group values. **(E)** Left: representative confocal images of age-matched and sex-matched fibroblasts from a healthy control patient and fibroblasts from a PD patient transfected with $PH_{PLC\delta1}$-RFP. Right: quantification of PM/cytoplasm intensity ratio of $PH_{PLC\delta1}$ in fibroblasts. PD group values are normalized to control group values. **(F)** $PI(3,4,5)P_3$ biosensor. Left**:** schematic of biosensor and lipid substrate. Middle: HEK293T cells expressing BTK_{PH}-GFP with or without α-Syn^{A53T}. Right: quantification. **(G)** Same as (F) only PI(3)P biosensor. **(H)** Left: Schematic of $PI(4,5)P_2$ biosensor. Right: quantification of $PH_{PLC\delta1}$ distribution under control or fibril conditions. Error bars represent the standard error of the mean. Statistical analysis were students t-test. * $p < 0.05$; ** $p < 0.01$; *** $p < 0.001$.

Figure S2

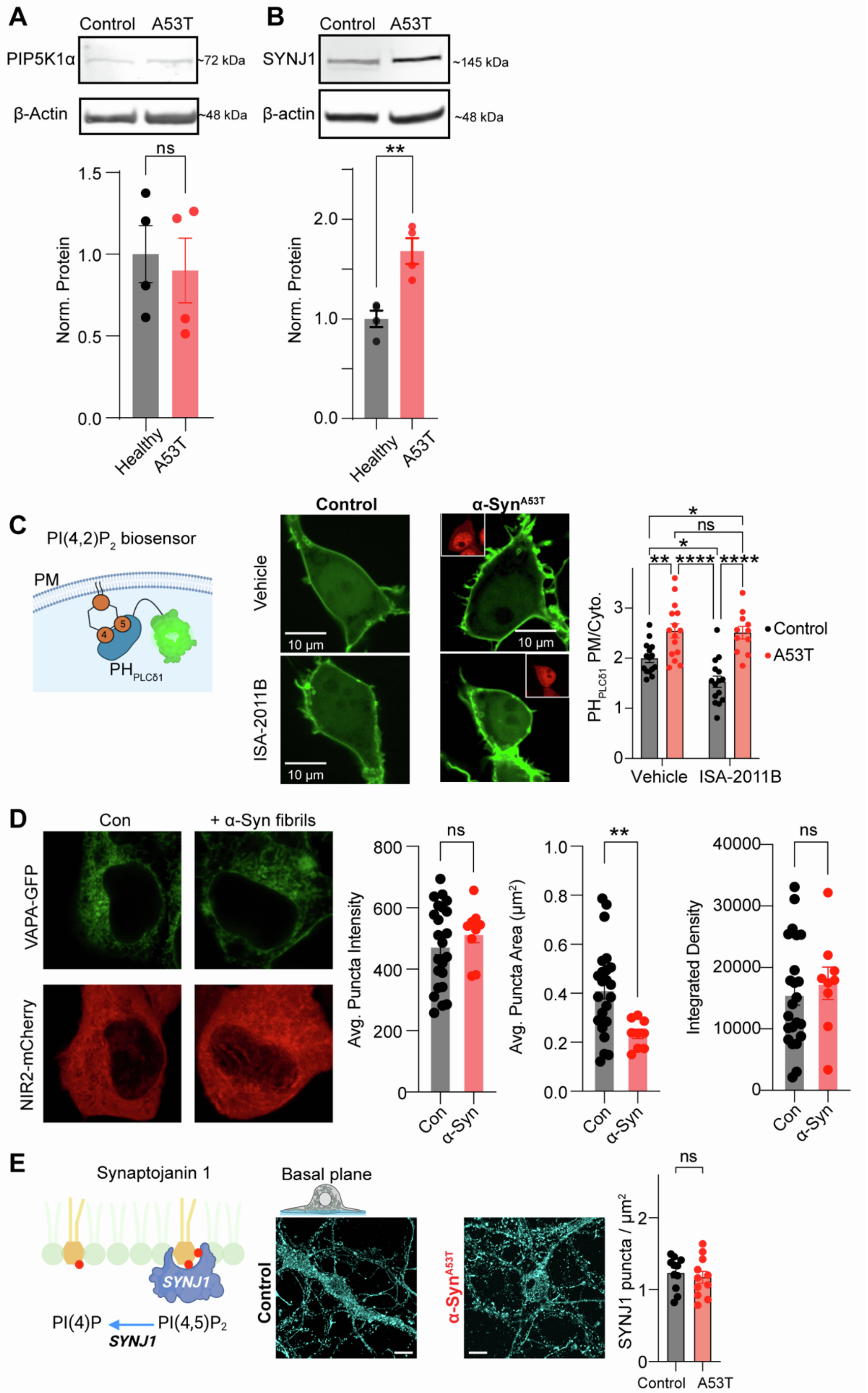

A
Control A53T
PIP5K1α
~72 kDa
β-Actin
~48 kDa
ns
Norm. Protein
Healthy
A53T
B
SYNJ1
~145 kDa
β-actin
~48 kDa
**
C
PI(4,2)P2 biosensor
PM
PHPLCδ1
Control
α-SynA53T
Vehicle
ISA-2011B
10 µm
PHPLCδ1 PM/Cyto.
Control
A53T
D
Con
+ α-Syn fibrils
VAPA-GFP
NIR2-mCherry
Avg. Puncta Intensity
Avg. Puncta Area (µm²)
Integrated Density
α-Syn
E
Synaptojanin 1
SYNJ1
PI(4)P
PI(4,5)P2
Basal plane
SYNJ1 puncta / µm²

Figure S2. α-Syn-dependent increases in PM PI(4,5)P_2 does not appear to involve PIP5K1α, Synaptojanin 1, or increased recruitment of NIR2. (A) Representative western blots from control and α-Syn^{A53T} fibroblast lysates probed for PIP5K1α and b-actin. **(B)** Same as (A) only synaptojanin 1. **(C)** Left: schematic of PI(4,5)P_2 biosensor, $PH_{PLCδ1}$. Middle: representative confocal images from HEK293T cells expressing $PH_{PLCδ1}$ with or without α-Syn^{A53T} expression. Right: quantification. **(D)** Left: Representative confocal images of control and α-Syn fibril-treated HEK293T cells transfected with VAPA-GFP and Nir2-mCherry. Right: Quantification of VAPA puncta intensity, area, and integrated density. **(E)** Left: Schematic and metabolism of synaptojanin-1. Middle: representative images of cortical neurons with or without α-Syn^{A53T} expression fixed and stained for anti- synaptojanin-1. Right: quantification. Error bars represent the standard error of the mean. Statistical analyses were students t-tests. NS = not significant; ns = not significant; * $p < 0.05$; ** $p < 0.01$; ** $p < 0.01$.

Figure S3

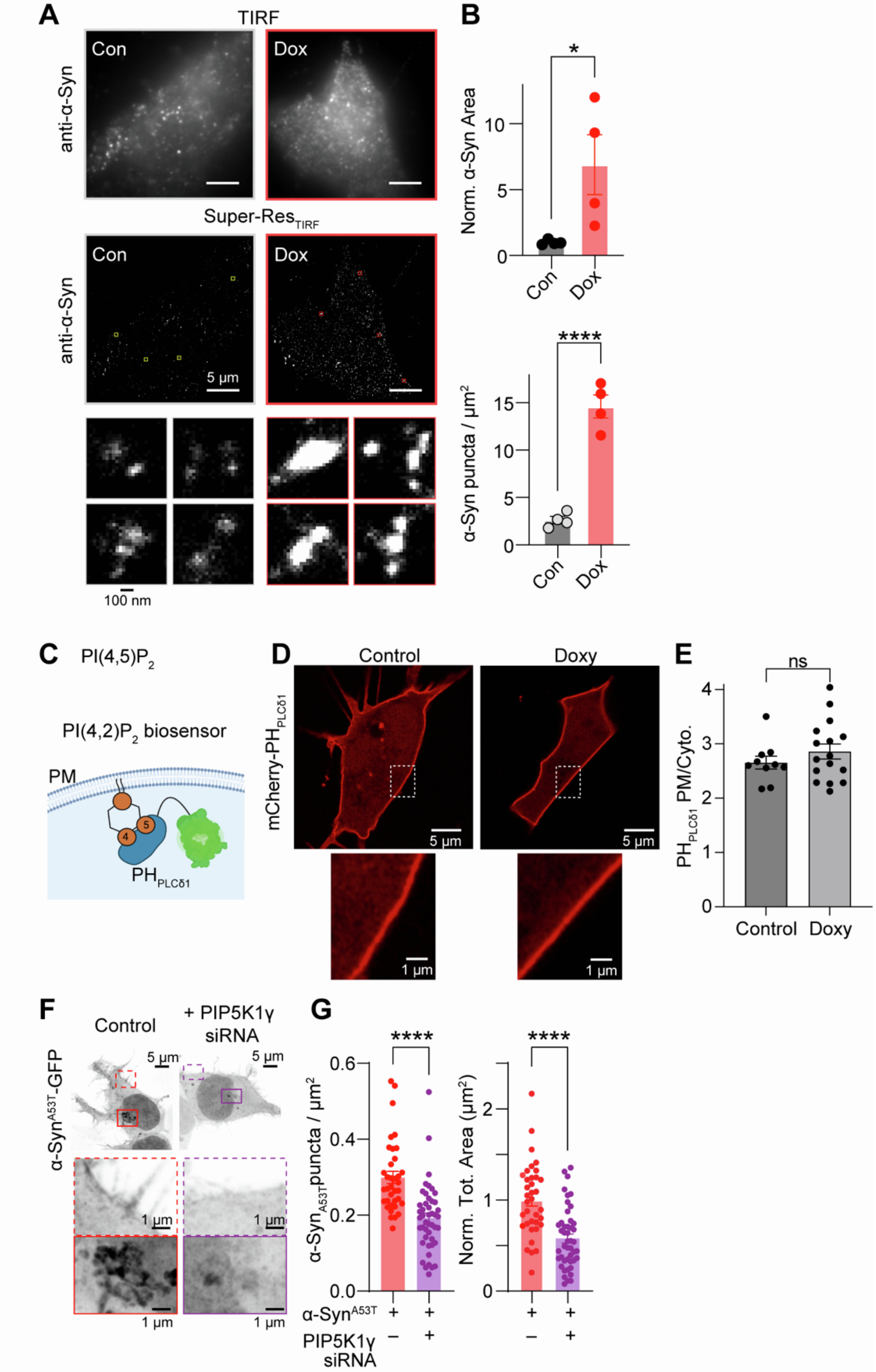

Figure S3. Knocking down PIP5K1γ decreases α-Syn aggregation. (A) Representative TIRF images and super-resolution localization maps (Super-Res$_{TIRF}$) of

undifferentiated control and doxycycline-induced α-Syn-overexpressing SH-SY5Y cells immunolabelled. Zoom images show selected α-Syn puncta. **(B)** Quantification of α-Syn staining in Super-Res$_{TIRF}$ images. Statistical analysis is students t-test. **(C)** Schematic of PH$_{PLC\delta1}$ binding to PI(4,5)P_2. **(D)** Representative confocal images from HEK293T cells expressing PH$_{PLC\delta1}$ treated with or without doxycycline to switch on α-Syn expression. **(E)** Quantification of PH$_{PLC\delta1}$ distribution. **(F)** Representative confocal images of control HEK293 cells and HEK293 cells transfected with siRNA PIP5K1γ. **(G)** Quantification of α-Syn^{A53T} density (left) and puncta area (right). Error bars represent the standard error of the mean. * $p < 0.05$; **** $p < 0.0001$.

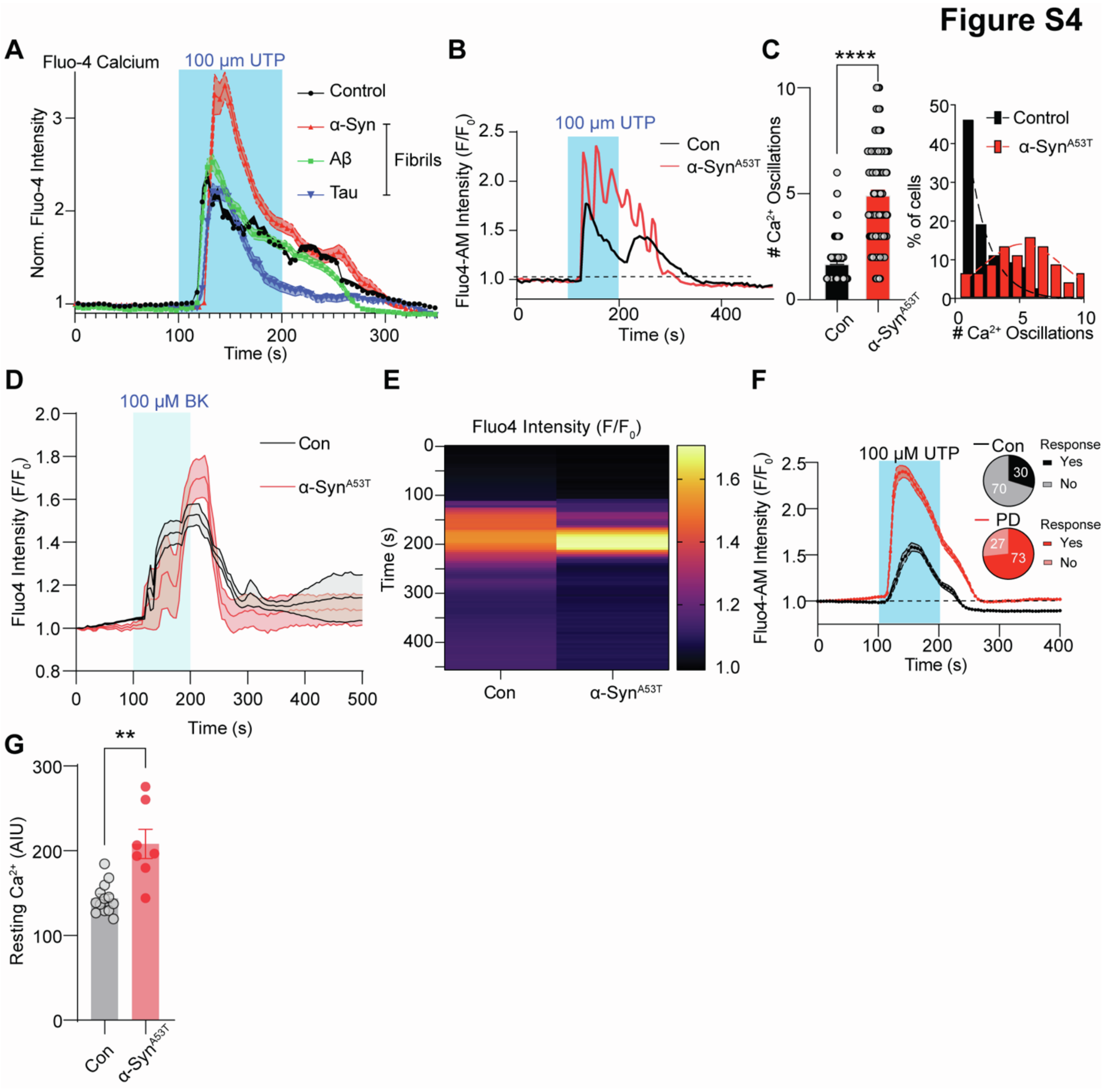

Figure S4. α-Syn fibrils and α-Syn^{A53T} augment IP$_3$-mediated Ca^{2+} release. (A) Quantification of normalized Fluo4-AM intensity in control and fibril treated cortical neurons perfused with 2 mM Ca^{2+} Ringer's solution before, during, and after application of 100 µM UTP) from 100 s to 200 s. Fluo4-AM intensity values are normalized to t = 0 s value. **(B)** Representative traces of cells transfected with or without α-Syn^{A53T}, loaded with Fluo-4, and treated with 100 µm UTP. **(C)** Quantification of Ca^{2+} oscillations (left) and proportion of cells that exhibit oscillations (right). **(D)** Quantification of normalized Fluo4-AM intensity in control and α-Syn^{A53T}-transfected mouse hippocampal neurons perfused

with 2 mM Ca^{2+} Ringer's solution before, during, and after application of 100 μM bradykinin (BK) from 100 s to 200 s. Fluo4-AM intensity values are normalized to t = 0 s value. **(E)** Heat map of Fluo4-AM assay in *(D)*. Heat map shows normalized Fluo4-AM intensity to initial recording in mouse hippocampal neurons. **(F)** Quantification of normalized Fluo4-AM intensity from patient fibroblasts during UTP perfusion. Pie charts show percentage of fibroblasts responding to UTP application. **(G)** Quantification of initial (t = 0 s) Fluo4-AM intensity recording in mouse hippocampal neurons from Fluo4-AM assay in *(D)*. Measurements were taken by selecting a cytosolic ROI to show resting Ca^{2+} levels at t = 0 s before BK application. Statistical analysis was student t-test. Error bars represent the standard error of the mean. ** $p < 0.01$ and **** $p < 0.0001$.

Figure S5

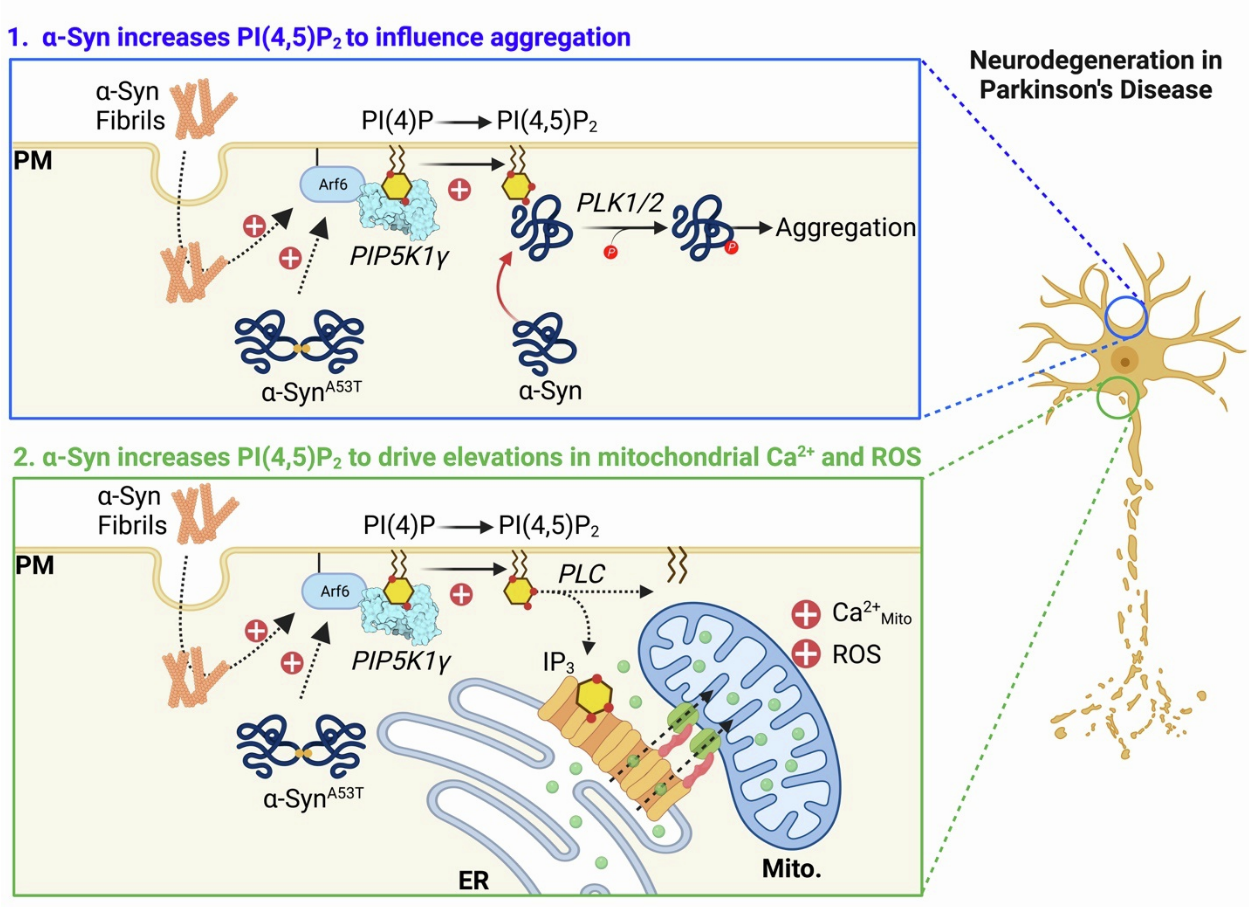

Figure S5. Model of how α-Syn fibrils and a-SynA53T mediate increases in plasma membrane PI(4,5)P2 to drive α-Syn aggregation and toxic elevations in mitochondrial Ca^{2+}.

References

1. Torpey, J.H., Meade, R.M., Mistry, R., Mason, J.M., and Madine, J. (2020). Insights Into Peptide Inhibition of Alpha-Synuclein Aggregation. Frontiers in neuroscience *14*, 561462. 10.3389/fnins.2020.561462.
2. Stojkovska, I., and Mazzulli, J.R. (2021). Detection of pathological alpha-synuclein aggregates in human iPSC-derived neurons and tissue. STAR Protoc *2*, 100372. 10.1016/j.xpro.2021.100372.
